# Does an apple a day keep away diseases? Evidence and mechanism of action

**DOI:** 10.1002/fsn3.3487

**Published:** 2023-06-20

**Authors:** Yue Zhang, Miao Zeng, Xiaolu Zhang, Qun Yu, Wenyun Zeng, Bin Yu, Jiali Gan, Shiwu Zhang, Xijuan Jiang

**Affiliations:** ^1^ School of Integrative Medicine Tianjin University of Traditional Chinese Medicine Tianjin China; ^2^ Department of Pathology Tianjin Union Medical Center Tianjin China; ^3^ School of International Education Tianjin University of Chinese Medicine Tianjin China

**Keywords:** apple, apple product, bioactive compound, disease, human health

## Abstract

Apples and their products exemplify the recently reemphasized link between dietary fruit intake and the alleviation of human disease. Their consumption does indeed improve human health due to their high phytochemical content. To identify potentially relevant articles from clinical trials, some epidemiological studies and meta‐analyses, and in vitro and in vivo studies (cell cultures and animal models), PubMed was searched from January 1, 2012, to May 15, 2022. This review summarized the potential effects of apple and apple products (juices, puree, pomace, dried apples, extracts rich in apple bioactives and single apple bioactives) on health. Apples and apple products have protective effects against cardiovascular diseases, cancer, as well as mild cognitive impairment and promote hair growth, healing of burn wounds, improve the oral environment, prevent niacin‐induced skin flushing, promote the relief of UV‐induced skin pigmentation, and improve the symptoms of atopic dermatitis as well as cedar hay fever among others. These effects are associated with various mechanisms, such as vascular endothelial protection, blood lipids lowering, anti‐inflammatory, antioxidant, antiapoptotic, anti‐invasion, and antimetastatic effects. Meanwhile, it has provided an important reference for the application and development of medicine, nutrition, and other fields.

## Graphical abstract

Apples and apple products (juices, puree, pomace, dried apples, extracts rich in apple bioactives and single apple bioactives) have protective effects against cardiovascular diseases and cancer, as well as cognitive functions and promote hair growth, healing of burn wounds, improve the oral environment, prevent niacin‐induced skin flushing, promote the relief of UV‐induced skin pigmentation, and improve the symptoms of atopic dermatitis as well as cedar hay fever among others. These effects are associated with various mechanisms, such as antioxidative, protecting vascular endothelium, reducing blood lipids, as well as anti‐inflammatory, pro‐/antiapoptotic, anti‐invasion, and metastasis effects.

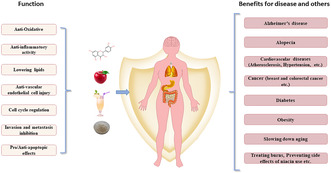

## 1 INTRODUCTION

The recommended daily fruit and vegetable allowance—as per the World Health and Food and Agriculture Organizations—is 400 grams (Falcomer et al., 2019) as a high intake of fruits and vegetables is associated with low all‐cause mortality rates (Hodgson et al., 2016; Wang et al., 2014). A 15‐year longitudinal study on the relationships between apple consumption and all‐cause mortality in 1456 women who were over 70 years of age showed that those who ate an apple a day were 35% less likely to die from any cause of death than those with low apple consumption (<5 g/d) (Hodgson et al., 2016). Relatedly, an observational study of 10,054 people from different regions of Finland concluded that apple intake was beneficial in alleviating chronic diseases such as ischemic heart disease, lung cancer, and Type 2 diabetes mellitus (T2DM) (Knekt et al., 2002). Apples are members of the *Malus* genus in the *Rosaceae* family. As one of the most prevalent fruits worldwide, apples are popular for their nutritional value. Apples contain several biologically active substances with beneficial effects on human health, and the active ingredients are not the same in different types of apples around the world (Dong et al., 2016; Qin et al., 2017; Vrhovsek et al., 2004). In this regard, the polyphenols, vitamins, triterpenes, dietary fiber, and other active ingredients in apples can even alleviate diseases, as verified in many in vitro and animal experiments, and further multistage clinical trials. People generally choose to consume fresh apples, but products such as apple juice, apple cider vinegar (ACV), apple powder, and apple cider are also becoming popular. This paper systematically reviewed the apple functions and potential challenges of apples to further promote apple‐related research for their application and development in medicine and food.

## 2 BIOACTIVE COMPOUNDS IN APPLES

Macroelements from apples such as highly abundant potassium, nitrogen, phosphorus, calcium, and magnesium, and trace elements such as boron, zinc, iron, manganese, and aluminum (Bouderbala et al., 2016). These minerals are essential components of human tissues and have special physiological functions such as the activation of various enzymes (Punchay et al., 2020).

The vitamin C of apples inhibits low‐density lipoprotein (LDL) oxidation, stabilizes the endothelium (Dalton et al., 2003; Lee et al., 2002), and lowers cholesterol levels, thus preventing diabetes and hypertension (Chambial et al., 2013). Moreover, vitamin C protects the skin from UV‐induced photodamage (Pullar et al., 2017; Wang, Jiang, et al., 2018) and prevents several cancers (Cieslak & Cullen, 2015; Harris et al., 2013; Hoffer et al., 2015; Monti et al., 2012). Other beneficial vitamins contained in apples are vitamins A and E, thiamin, riboflavin, nicotinic acid, folic acid, and pyridoxine (Islam et al., 2016).

**FIGURE 1:**
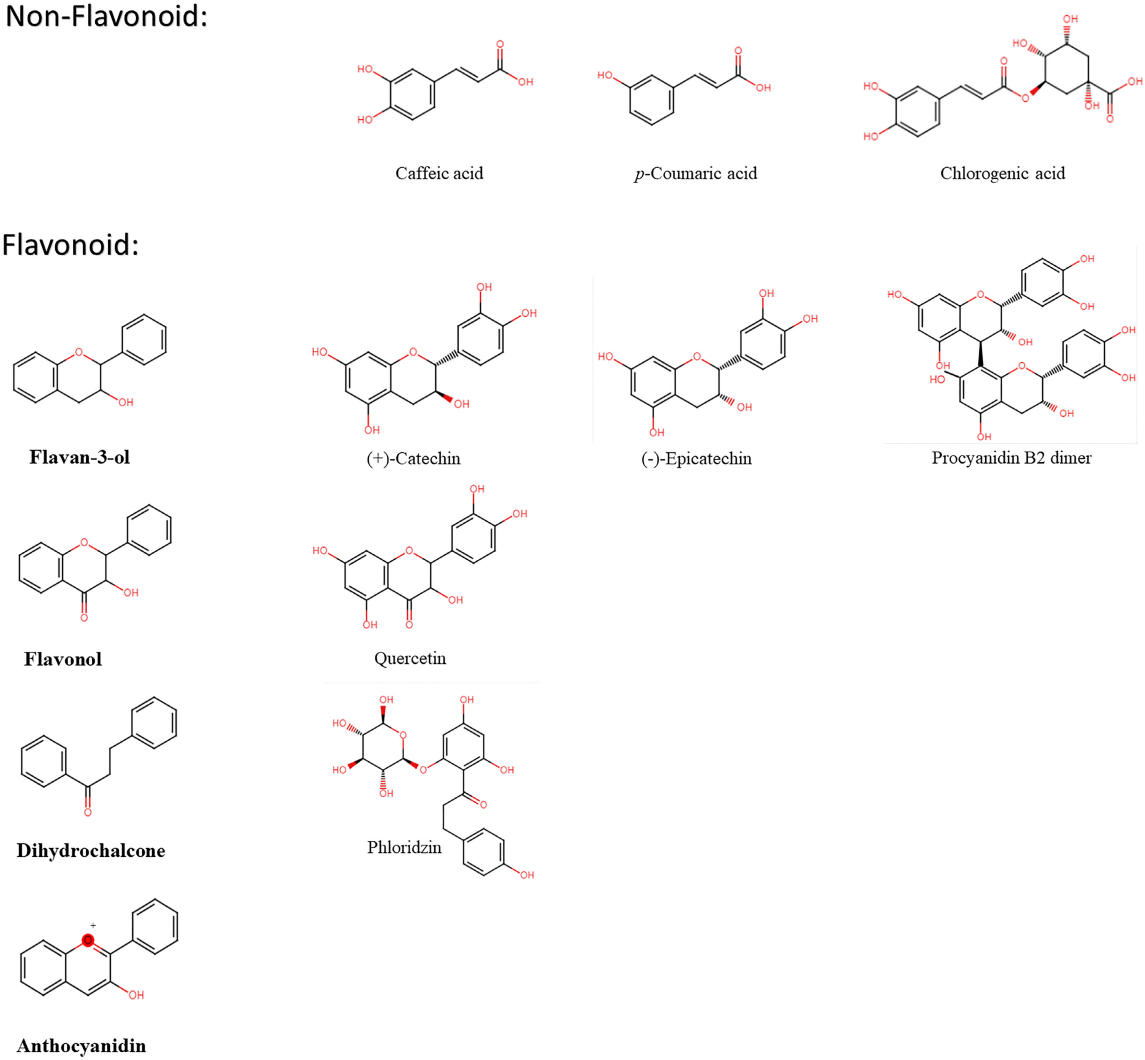
Chemical structures of representative phenolic compounds in apple fruits.

The bioactivities of apple polyphenols include anti‐inflammatory, antioxidant, and antiviral properties (Matos et al., 2020), inhibition of platelet‐activated aggregation (Holt et al., 2006; Hubbard et al., 2003; Ravishankar et al., 2018; Vaiyapuri et al., 2013, 2015), and neuroprotection (Jensen et al., 2014). Moreover, they prevent cardiovascular diseases (CVDs) (Heiss et al., 2010; McCullough et al., 2012; Ponzo et al., 2015), degenerative diseases, and enteritis, and protect the gastric wall from aspirin (Paturi et al., 2014), and the colonic mucosa in cases of ulcerative colitis (D'Argenio et al., 2012). From high‐performance liquid chromatography–high‐resolution mass spectrometry analyses, 12 representative apple phenolic compounds were identified (Antonic et al., 2020; Feng et al., 2021) (Table 1, Figure 1), 80% of which were contained in the peels (Leccese et al., 2009). Polyphenolic phytochemicals are classified as flavanols, catechins (or flavanols), flavonols, flavanones, isoflavones, and anthocyanins. Phloretin is a natural polyphenolic compound, which is known as a glucose transporter (GLUT) inhibitor (Takeno et al., 2018). Phlorizin, the major dihydrochalcone flavonoid in apples, is known to inhibit renal glucose reabsorption and regulate blood glucose levels (Rosenwasser et al., 2013), mainly found in the apple peel (Vrhovsek et al., 2004).

**TABLE 1:** The concentration of representative phenolic compounds in different parts and different forms.

Compound	Different forms			
	Dried apple pomace (mg/kg)	Whole apple (mg/kg)	Fresh juice (mg/L)	Commercial juice (mg/L)
Nonflavonoid				
Caffeic acid	3–280	15–2960		
*p*‐Coumaric acid		4–260		
Chlorogenic acid				
Flavonoid				
Flavan‐3‐ol		4622–25,480	50–393	14–124
(+)‐Catechin	1–127			
(−)‐Epicatechin	4.2–640	69–2760		
Procyanidin B2 dimer	48.8–590.2	69–2000		
Flavonol		80–1660	0.4–27	4–14
Quercetin	69–373.8			
Dihydrochalcone		49–434	10–171	9–87
Phloridzin	8–1435.4			
Anthocyanidin		10–551		

Bioactive compounds from apples include triterpenoids (McGhie et al., 2012), of which ursolic and oleanolic acids account for 79%–95%. Triterpenoids have anticancer, anti‐inflammatory, antihyperlipidemia, hypoglycemic, analgesic, gastric and liver protective, antiulcer, and antiatherosclerosis activities (López‐Hortas et al., 2018). Notably, the anticancer properties (Shi et al., 2017) of ursolic acid that was isolated from apple peels specifically suppressed the proliferation of various tumor cell lines (Ma et al., 2005).

Apples are composed of both soluble and insoluble dietary fibers (Gidley & Yakubov, 2019; Han et al., 2017; Verma & Banerjee, 2010), with the peel containing about 2–3 times as much fiber as the pulp. Dietary fiber promoted intestinal health (Carvajal‐Millan et al., 2005), prevented CVDs (King et al., 2012), and reduced mortality rates (Barber et al., 2020). A major component of apple dietary fibers, pectin, had excellent detoxification properties via the binding of harmful metals like lead and mercury (Patocka et al., 2020). Furthermore, pectin delayed gastric emptying (Di Lorenzo et al., 1988; Schwartz et al., 1982), prevented obesity, and lowered blood lipids (Brouns et al., 2012; González‐Estrada et al., 2015).

## 3 THE BENEFICIAL HEALTH EFFECTS OF APPLES

### 3.1 Apples and cardiovascular disease

About 23.6 million mortalities from CVDs (Roth et al., 2020) including coronary artery, cerebrovascular, and peripheral arterial diseases are predicted by 2030 (Stewart et al., 2017). Increased oxidative stress (Mishra et al., 2020), endothelial dysfunction (Zhang et al., 2016), metabolism disorders (Agwa et al., 2021), and inflammation (Zhang et al., 2018) are all associated with the development and pathogenesis of CVDs. We summarized the evidence that apples improve cardiovascular parameters in humans through their antioxidant, antiendothelial damage, lipid‐lowering, and anti‐inflammatory effects, extensively analyzed by animal and in vitro experiments to explain the underlying mechanisms.

#### 3.1.1 Antioxidant properties

A good balance between reactive oxygen species (ROS) and antioxidants is essential for cardiovascular function (Ali et al., 2020). Oxidative stress leads to protein oxidation, lipid peroxidation (LPO), and DNA damage (Kreuzer et al., 2020), ultimately leading to CVDs (Cervantes Gracia et al., 2017). Frequent consumption of foods rich in antioxidants can reduce the risk of CVD by up to 30% (Rimm et al., 1996). Apples and apple juice contain numerous antioxidants that may contribute to CVD prevention (Boyer & Liu, 2004; Prior & Cao, 2000). Fresh fruits, especially apples, are a key source of natural antioxidants, like polyphenols, vitamins C (Anjum et al., 2020), and flavonoids (Bondonno et al., 2012; Molagoda et al., 2021).

##### Clinical trials

The antioxidant capacity of apples is largely attributed to polyphenols and vitamins, but there is considerable debate as to which of these is most important. A large number of literature provided evidence that polyphenols are the main contributors to the antioxidant capacity of apples or apple juice (Gardner et al., 2000; Gliszczynska‐Swiglo & Tyrakowska, 2003; Tsao et al., 2005). In 23 healthy participants, consumption of apple juice increased total plasma antioxidant capacity as well as erythrocyte glutathione peroxidase (GSH‐Px) and catalase (CAT) activities and decreased plasma malondialdehyde (MDA) levels. Moreover, apple phenolic compounds were the main inhibitors of lipid oxidation (Yuan et al., 2011). Meanwhile, the single rapid consumption of 150 mL of apple juice in healthy volunteers also resulted in good antioxidant activity (Ko et al., 2005). Interestingly, 12 healthy volunteers consumed either a liter of apple juice with/without polyphenols or cloudy apple juice. Consumption of all three juices increased within an hour; the ferric‐reducing ability and 2,2‐diphenyl‐1‐picrylhydrazyl radical scavenging capacity of plasma and serum, respectively, reflecting enhanced plasma antioxidant capacities. While plasma polyphenol concentrations did not increase, this change was also shown to be associated with an increase in fructose‐induced uric acid (Godycki‐Cwirko et al., 2010). A clinical trial found that consumption of polyphenol‐rich apple juice did not significantly change plasma antioxidant activity, while consumption of vitamin‐C‐rich apple juice significantly reduced total glutathione (GSH) levels in peripheral blood (Soriano‐Maldonado et al., 2014). It has also been reported that consuming apple juice with natural antioxidant vitamin C and polyphenols may have modest beneficial effects on cardiometabolic markers compared to apple polyphenols alone (Soriano‐Maldonado et al., 2014). Taken together, apple juice undoubtedly improves indicators of oxidative stress, but it is unclear whether this is the result of a single compound or a synergistic effect of all the compounds in the apple.

##### Animal and cellular studies

Several animal studies have evaluated the antioxidant capacity of apple products, such as dehydrated apples, ACV, apple puree, and phenolic extracts. Apples and their extracts improve oxidative stress indicators and thus alleviate atherosclerosis. For example, Pailar et al. showed that dehydrated apple products significantly reduced oxidative stress markers including carbonyl groups and 8‐hydroxydeoxyguanosine (8‐OHdG) in a TAM‐induced rat model of oxidative stress (Codoñer‐Franch et al., 2013). Relatedly, the consumption of ACV had good efficacy against antioxidant stress. Consumption of ACV inhibited obesity‐induced oxidative stress in Wistar rats fed on high‐fat diets, describing a decrease in MDA levels but an increase in thiol group concentrations, superoxide dismutase (SOD), GSH‐Px, and CAT activities (Halima et al., 2018). Furthermore, either apple puree or phenolic extracts reduced the levels of 4‐hydroxy‐2‐nonenal (4‐HNE), a potent toxic LPO end product associated with endothelial dysfunction and atherosclerosis, in the gastrointestinal tract of atherosclerotic mice (Bolea et al., 2021). The beneficial effect observed may be attributed to the high levels of phenolic proanthocyanidins present in apples. Similar results were obtained in vitro studies (Boléa et al., 2019). In vitro studies showed that polyphenols in apples have antioxidant properties that inhibit lipid oxidation during gastric digestion (Boléa et al., 2019). These polyphenols achieved this by reducing the pro‐oxidative form of iron myoglobin (MbFeIV = O) to myoglobin (MbFeIII) through the catechol nucleus. This process released free iron and prevented lipid oxidation from occurring (Bolea et al., 2021; Lorrain et al., 2010). Apple polyphenol intervention protected Caco‐2 cells from chronic high‐glucose‐induced oxidative stress, increased the total antioxidant capacity and GSH levels, and reduced glyoxalase I activity, mainly via the glyoxalase pathway (Cianfruglia et al., 2020).

#### 3.1.2 Endothelial protection effects

Vascular endothelial cells play a key role in maintaining vascular integrity, transmitting vascular information, and secreting vasoactive substances (Chavez et al., 2011; He et al., 2021). Vascular endothelial dysfunction is associated with the development of CVDs, such as coronary artery and cerebrovascular diseases and hypertension (Li et al., 2018; Ren et al., 2020; Zhang et al., 2019). The balance between endothelial injury and recovery is essential for the maintenance of endothelial function (Shantsila et al., 2007). The polyphenolic component of apples was found to be the main active component exerting endothelial protection.

##### Clinical trials

A single‐center, double‐blind randomized crossover trial investigated the effect of oral administration of apple polyphenol extracts on endothelial function in 60 patients with either borderline (bp 130–139/85–89 mm Hg) or mild hypertension (bp 140–165/90–95 mm Hg). For 4 weeks, patients received either an apple polyphenol extract or a placebo. Intake of the extracts resulted in significant acute improvements in flow‐mediated dilation of the brachial artery, an indicator of endothelial function of all hypertension patients (Saarenhovi et al., 2017). Meanwhile, the consumption of flavonoid‐rich apples improved NO status and enhanced endothelial function (Bondonno et al., 2012). In a crossover clinical trial of 30 participants (Bondonno et al., 2018) with at least either a CVDs or its risk factor—mild‐to‐moderate hypertension, hyperglycemia, elevated fasting cholesterol, and central obesity—participants consumed either entire Cripps Pink apples with peels (high flavonoid) or Cripps Pink apples blended with water (low flavonoid) for four consecutive weeks. High flavonoid intake leads to a significant increase in flow‐mediated dilation and suggests that it is better to eat apples with their peels.

##### Animals and cellular studies

Jia et al. showed that apple peel polyphenol extracts reduced AST and ALT activities, elevated serum NO and 6‐Keto‐PGF1a levels, and lowered serum ET‐1 and TXB2 levels in high‐choline‐fed mice (Jia et al., 2017). Similar effects were observed with interventions of phloretin (Ren et al., 2016). Apple extract (AE) elevated nitrite production, and increased wound closure of endothelial cells damaged by H_2_O_2_ (Wattanapitayakul et al., 2021). AE treatments had a protective effect on human umbilical vein endothelial cells (HUVEC) exposed to cytotoxic glycosylated protein GFBS/FeCl_3_. This effect was demonstrated by dose‐dependent reduction in LPO, cytochrome c (Cyto C) reductase, and glutathione S‐transferase (GST) to near‐normal levels (Nishigaki et al., 2010). Relatedly, apple polyphenol treatments significantly inhibited the ROS/MAPK/NF‐κB signaling pathway, which in turn reduced the expression of monocyte chemotactic protein‐1 (MCP‐1), intercellular adhesion molecule‐1 (ICAM‐1), vascular cell adhesion protein‐1 (VCAM‐1), and monocyte adhesion to rat aortic endothelial cells (Xu et al., 2015).

#### 3.1.3 Lipid‐lowering effects

As dyslipidemia is a major risk factor for CVD and premature atherosclerosis, atherogenic lipoproteins are well‐established targets for CVD risk reduction interventions (Gaudet et al., 2017). The intake of apples, apple products, and apple polyphenols decreased atherogenic lipoproteins such as total cholesterol (TC), triglycerides (TGs), and low‐density lipoprotein cholesterol (LDL‐C).

##### Clinical trials

The CVD risk factors, such as cholesterol, drastically increase after menopause due to ovarian hormone deficiency (Gidding, 2016). Premenopausal women thus have a lower incidence of CVD than men. In a 1‐year clinical trial study, the reduction in CVD risk factors in postmenopausal women through dried apple consumption was evaluated. After 3 months, women who consumed dried apples had a significant reduction of 9% and 16% in serum TC and LDL‐C levels, respectively, when compared to the baseline. After 6 months, these levels further reduced by 13% and 24% but thereafter remained the same (Chai et al., 2012). Among the diverse AE, the specific compound that has the best effect on lowering blood lipids remains enigmatic. Another randomized controlled trial in volunteers with mild hypercholesterolemia demonstrated that consuming two anthocyanin‐rich apples per day lowered serum TC, LDL‐C, and TG (Koutsos et al., 2020). Gitter et al. showed that healthy volunteers who for 20 weeks consumed whole apples, apple pomace (AP), and cloudy apple juice with no additives lowered their serum LDL‐C by 6.7%, 7.9%, and 2.2%, respectively. Notably, those who consumed apple juice had the least reduction in LDL‐C levels (Ravn‐Haren et al., 2013). However, the consumption of clear apple juice had a significant adverse effect on lipids, with a significant increase in both TC and LDL‐C concentrations after 4 weeks of drinking 500 mL of clear apple juice per day. Hence, the consumption of either whole apples or apple puree, but not clear apple juice, is recommended for lowering LDL‐C levels.

The hypolipidemic effect of apples is possibly due to polyphenols. The polyphenolic compound content in apples can be significantly increased by fermentation (Liu et al., 2021) as bacterial hydrolases release polyphenols from fibers, thus enhancing their intestinal bioavailability. Indeed, in a study where either fermented or unfermented Anurka apple puree was administered to volunteers for 8 weeks (after a 4‐week run‐in period), which was succeeded by 4 weeks of follow‐up visits, those who consumed malolactic fermented pure had higher serum high‐density lipoprotein cholesterol (HDL‐C) than those who consumed unfermented puree (Tenore et al., 2019).

##### Animal and cellular studies

Apples and apple products have been found to have a positive impact on blood lipid levels, which in turn reduces the risk of CVD (D'Assante et al., 2021). AP and apple juice concentrate treatments reduced body and white fat weight, and atherosclerotic lesion scores in obese rats on high‐fat diets (HFD) (Cho et al., 2013). In a study conducted on Sprague–Dawley rats fed with apples, it was observed that supplementation of their diet with apple polyphenols led to an increase in the secretion of bile acids and neutral steroids in their feces. It was also found that the levels of TC in both the liver and serum decreased. Further investigation revealed that this effect was due to the metabolites of (−)‐epicatechin and catechin monomer, which modulate hepatic cholesterol 7α‐hydroxylase activity and increase steroid excretion (Osada et al., 2006). Relatedly, apple polyphenols induced inguinal white adipose browning and reduced adipose tissue mass in obese mice by activating the peripheral catecholamine synthesis FGF21‐PGC‐1α cascade (Tamura et al., 2020). Jia Tian et al. showed that polyphenolic extracts from apple peels (PAP) had better lipid‐lowering and cardioprotective effects than polyphenolic extracts from the apple flesh (PAF) due to the significantly higher phenolic and flavonoid content in the PAP (Tian et al., 2018). Phenols and flavonoids in apples are hypothesized as the main compounds that confer cardiovascular protection. In a study conducted by Serra et al., the serum lipid profiles of three different types of apples with varying concentrations of phenolics and fiber were evaluated in HFD Wistar rats. The results showed that apples with higher concentrations of catechin, epicatechin, procyanidin B1, and β‐carotene significantly reduced the serum levels of TG, TC, and LDL‐C (Serra et al., 2012).

Anurka apples, which are native to southern Italy, are known to have the highest concentration of polyphenols among all apple varieties (Tenore et al., 2013). Indeed, Gian et al. showed that consumption of this cultivar reduced TC and LDL‐C levels by 8.3% and 14.5%, respectively, and increased those of HDL‐C by 15.2% (Tenore, Caruso, Buonomo, D'Urso, et al., 2017). A monocentric, double‐blind, placebo‐controlled 12‐week study on 250 mildly hypercholesterolemic subjects (Tenore, Caruso, Buonomo, D'Avino, et al., 2017) evaluated the effect of Anurka apple polyphenol extracts on serum lipids via daily consumption of two capsules of either Anurka apple polyphenol extract or a placebo for 4 weeks. Treatment with Anurka apple polyphenol extract decreased TC and LDL‐C levels by 24.9% and 37.5%, respectively, and increased HDL‐C levels by 49.2%. Remarkably, the effect of LDL‐C lowering was similar to that of simvastatin (40 mg) or atorvastatin (10 mg). This study investigated the metabolism of human hepatocytes treated with Anurka polyphenol extract using isotope labeling and high‐resolution mass spectrometry in vitro (Sommella et al., 2019). These extracts enhanced mitochondrial respiration in liver cells, thereby inhibiting the use of citrate for lipogenesis. This leads to a reduction in the synthesis of cholesterol and fatty acids.

#### 3.1.4 Anti‐inflammatory effects

Apple, phloretin, quercetin, and apple polysaccharides consumption have an anti‐inflammatory effect that lowers chronic low‐grade systemic inflammation such as atherosclerosis and hypertension (Wang, Kang, et al., 2018).

##### Clinical trials

Consumption of dried daily portions of the red‐fleshed or white‐fleshed apple for 2 weeks by 30 healthy volunteers reduced the expression of proinflammatory genes in peripheral blood mononuclear cells (PBMCs) (Sommella et al., 2019). In a randomized, parallel‐arm intervention clinical trial on the effect of whole Gala apples on inflammatory markers in plasma and PBMCs, 44 participants consumed either three whole Gala apples per day or none for 6 weeks. Apples decreased plasma levels of C‐reactive protein (CRP), interleukin‐6 (IL‐6), and LPS‐binding protein (LBP) by 17%, 12.4%, and 20.7%, respectively. PBMC of volunteers secreted 28.3% and 11.0% less IL‐6 and IL‐17, respectively, than those of the control group (Liddle et al., 2021). That apple consumption was closely related to low serum CRP concentration was corroborated by yet another study (Chun et al., 2008). Thus, the anti‐inflammatory effect of apples is supported by numerous clinical trial studies.

##### Animal and cellular studies

The anti‐inflammatory effects of polyphenols result from not only their antioxidative activity but also their modulation of cell signaling cascades involved in the production of inflammatory cytokines (Joseph et al., 2016). From 109 apple cultivars, those rich in proanthocyanidin were the most effective in inhibiting nuclear factor‐κB (NF‐κB), whereas triterpenes‐rich cultivars repressed the promoter of the tumor necrosis factor‐alpha (TNFα) gene (Andre et al., 2012). A red‐fleshed genetically engineered variant of “Royal Gala” apples (flavonoid enriched) not only reduced inflammatory biomarkers but also positively modulated the colonic microbiota in rats, which was strongly associated with low‐grade inflammation (Espley et al., 2014). Moreover, phloretin is known to scavenge methylglyoxal to block advanced glycosylation end products (AGEs), and it has been shown to exert its anti‐inflammatory effects via inhibiting the RAGE/p38 MAPK/NF‐κB signaling pathway (Zhou, Gong, & Wang, 2019). Phloretin was found to decrease the expression of proangiogenic factors (such as leptin, IL‐1β, IL‐6, MCP‐1, and NF‐κB) in adipocytes stimulated with LPS and CoCl_2_. This was achieved, in part, by activating the peroxisome proliferator‐activated receptor γ (Liddle et al., 2020). Quercetin reduced atherosclerotic plaque size in atherosclerotic mice via a high fructose diet and inhibited LPS‐induced inflammatory responses that were mediated by the phosphoinositide 3‐kinase (PI3K)/AKT pathway (Lu et al., 2017). CVD is closely related to dysbiosis (Serena et al., 2018) and as apple polysaccharides significantly attenuated intestinal permeability and chronic inflammation in mice on HFDs (Wang et al., 2017), they possibly contribute to a healthy gut microbiome. In the same mice, apple polysaccharides upregulated the expression of occludin, and reduced plasma levels of LBP, TNFα, MCP‐1, CXCL‐1, and IL‐1β. Taken together, the anti‐inflammatory effect of various apple polyphenols has been corroborated both by in vitro and in vivo studies. These results provide a solid platform for future clinical research to determine whether patients suffering from inflammatory diseases may benefit from consuming proanthocyanidin‐rich apples, triterpenes‐rich apples, or other apple products.

### 3.2 Apples and diabetes

Diabetes mellitus is not only an endocrine system disorder with a complicated pathogenesis (Nathan et al., 1993) but is also only behind cancer and CVDs as the leading cause of death worldwide (Yoon et al., 2021). As postprandial hyperglycemia is a significant independent risk factor that contributes to complications associated with T2DM (Cavalot et al., 2006), its control is recommended (Abuelizz et al., 2019). One way is by optimizing the functionality of foods. Apples and their components (McDougall et al., 2005; Schulze et al., 2014) are considered good choices since they slow starch and disaccharide digestion, thus delaying glucose absorption after a carbohydrate‐containing meal or beverage.

The consumption of dried apples reduced postprandial blood glucose and improved insulin response in 21 healthy individuals (Sansone et al., 2018). Similarly, the effect of prior administration of AE was evaluated both in 10 healthy male volunteers and C57 BL/6N mice. Consumption of the AE before an oral glucose tolerance test (OGTT) by the volunteers reduced venous blood glucose and insulin levels, which was consistent with the murine experiment. The findings of this experiment also confirmed that AE, particularly phlorizin, decreased glucose uptake mediated by sodium‐coupled glucose transporter protein‐1 (SGLT1) (Schulze et al., 2014). Relatedly, the antihyperglycemic activity of a low‐sugar, fiber‐ and phloridzin‐rich powder from unripe apples was assessed on six women at risk of diabetes via 50 g OGTTs. Acute consumption of the AE improved glucose metabolism by a twofold reduction in the postprandial glucose response and a fivefold increase in urinary glucose excretion (Makarova et al., 2015).

In a 12‐week study, 65 participants with fasting blood glucose (FPG) levels of 100–125 mg/dL randomly received either AP‐containing tablets or a placebo. Long‐term administration of AP significantly reduced postprandial blood glucose (Shoji et al., 2017), validating the postprandial blood‐glucose‐lowering effect of apples in healthy people. However, this effect was yet to be confirmed in patients with impaired FPG. Vinegar intake before bedtime improved insulin resistance and fasting blood sugar levels in both healthy participants and patients with diabetes. One study found that the intake of ACV has a good effect on diabetics (Johnston et al., 2010; Leeman et al., 2005). This was corroborated by a similar study on 70 participants with T2DM and hyperlipidemia who drank two tablespoons of ACV before lunch and dinner (Gheflati et al., 2019). In a double‐blind, randomized, placebo‐controlled cross‐over design clinical trial, 30 men with impaired fasting glucose consumed 500 mL of either apple juice treated with invertase and glucose oxidase/CAT, or untreated apple juice. Due to this enzymatic treatment, a reduction of 21%, 68%, and 47% of the sugar content, postprandial glycemic response, and venous serum insulin response, respectively, were observed. This reduced the glycemic load by 74.6% without any adverse gastrointestinal side effects (Laue et al., 2019). In a study of 2987 pregnant Chinese women to evaluate the relationship between mid‐term pregnancy consumption of fruit, vegetable, and fruit juice and the risk of gestational diabetes mellitus (GDM), apples reduced the incidence of GMD, but excessive intake of grape, melon, and potatoes contributed to GMD (Li et al., 2021). Taken together, apples and their components improve postprandial blood sugar in various populations.

Cloudy apple juice and apple peel extracts were beneficial to pancreatic LPO, antioxidant enzymes, and inflammatory status in diabetic rats (Fathy & Drees, 2016). Moreover, the polyphenol phloridzin blocked the absorption of glucose/galactose from the intestinal lumen into intestinal epithelial cells by inhibiting SGLT1 (Najafian et al., 2012). This explains the low postprandial blood sugar in individuals—who are candidates for insulin resistance—treated with apples and their extracts.

### 3.3 Apples and cancer

Chronic diseases are a global public health burden (Magliano et al., 2019). In 2020, cancer was second only to CVDs as the most common chronic disease, with an estimated 19 million new cases and 10 million deaths per year worldwide (Sung et al., 2021). Apple consumption reduced the risk of cancers, such as head and neck cancer, liver cancer, prostate cancer, colon cancer, and breast cancer (Fabiani et al., 2016). Furthermore, AE contributes to the prevention of tumors.

Prostate cancer (PCa) is second only to lung cancer as the most common cancer in men, with more than 1.2 million new cases diagnosed each year and more than 350,000 mortalities worldwide (Rebello et al., 2021). Apple consumption was negatively associated with the risk of PCa in a case–control study of 50 PCa patients and 100 healthy volunteers (Askari et al., 2014). Head and neck cancers (HNC), mainly caused by tobacco and alcohol consumption, are a major cause of global deaths (Patel et al., 2022). Consumption of fruits and vegetables has been independently associated with a reduced risk of HNC (Pavia et al., 2006). In a 6‐ to 7‐year prospective observational study of 490,802 participants to evaluate associations between fruit and vegetable consumption and HNC incidence, apple consumption significantly reduced the risk of HNC (Freedman et al., 2008).

In recent years, breast cancer incidences have been increasing, particularly among young women (Naik et al., 2020). Apple polyphenol extracts inhibited the proliferation and migration of human breast cancer MDA‐MB‐231 cells by reducing the expression of ubiquitin like with PHD and ring finger domains 1 (UHRF1), matrix metalloproteinase 2 (MMP2), DNA methyltransferase 3 alpha (DNMT3a), and DNMT3b (Song et al., 2017). Studies showed that extracts from Anurka apple, specifically polyphenols, inhibited AKT activation and downregulated oncoproteins such as NF‐kB, c‐myc, and β‐catenin. Furthermore, the extracts activated c‐Jun N‐terminal kinase (JNK) by inducing ROS production and were found to selectively kill triple‐negative breast cancer cells (Martino et al., 2019). Apple peel flavonoid fraction 4 (AF4) is an ethanol extract of the flavonoid‐rich Northern Spy apple cultivar (Loung et al., 2019). AF4 inhibited MDA‐MB‐231 cell proliferation by targeting cyclin‐dependent kinase 4 (CDK4) and cyclin D3. Additionally, AF4 has been shown to inhibit cell migration and invasion by targeting MMP2. Meanwhile, AF4 inhibited the phosphorylation of phosphatase and tensin homolog (PTEN) (Ser380) and decreased Thr308 phosphorylation‐induced Akt activation (Loung et al., 2019). In another study, apple dihydrochalcone phloretin effectively inhibited glucose starvation and chemotherapy‐induced cytoprotective autophagy in MCF7 and MDA‐MB‐231 cell lines by suppressing the mechanistic target of rapamycin (mTOR)/Unc‐51‐like kinase 1(ULK1) signaling pathway (Chen et al., 2021). Apple polyphenol phloretin complexed with ruthenium‐activated p53 to inhibit the intrinsic apoptotic process and regulate tumor invasion by downregulating the PI3K/AKT/mTOR pathway and MMP9 levels in breast cancer cells (Roy et al., 2022).

Colorectal cancer, a common digestive tract malignancy that is associated with increased morbidity rates (Deng et al., 2020), is the second leading cause of cancer‐related deaths worldwide. Persistent colonic inflammation is a major risk factor for colorectal cancer. In vitro and in vivo studies showed that upon the consumption of apple and berry fruits, colorectal cancer risks were reduced (Jaganathan et al., 2014), which was attributed to the effects of plant‐associated anticancer chemical components in apples. Modified apple polysaccharides were found to improve apoptosis resistance and inflammation state in the colonic mucosa by inhibiting the binding of galectin‐3 to its ligand and reducing the expressions of mucin 1 (MUC1), respectively (Li et al., 2012; Sun et al., 2018). In vitro experiments showed that apple polysaccharides mildly upregulated toll‐like receptor 4 (TLR‐4) signaling and induced macrophage maturation toward the M1 phenotype (Sun et al., 2020). Further research showed that apple oligogalactan inhibited HT‐29 and SW‐620 colon cancer cell growth via the TLR‐4/NF‐κB pathway (Li et al., 2017).

Liver cancer ranks fifth in terms of prevalence among cancers in Asia and is the second most common cause of death from malignant tumors (Zhang et al., 2022). High expression of GLUT2 was observed in both human hepatocellular carcinoma samples and HepG2 cells and phloretin was found to be an effective inhibitor of GLUT2. Studies have demonstrated the potential of phloretin in the treatment of hepatocellular carcinoma. Yang et al. (2009) demonstrated that the combination of phloretin and paclitaxel (PTX) enhanced the antitumor activity of PTX and desensitized hepatoma cells to the chemotherapy drug. Furthermore, the enhancement of PTX‐induced apoptosis in human hepatocellular carcinoma cells by phloretin involved the activation of caspases‐3, 8, and 9. In HepG2 cells, AF4 treatment caused inhibition of cell growth in a time‐ and dose‐dependent manner. It also induced a block in the G2/M phase and activated caspase, leading to apoptosis. Intriguingly, AF4 demonstrated stronger antiproliferative effects in vitro compared to sorafenib and showed potential as a natural chemotherapeutic agent for cancer treatment (Wu et al., 2009).

Quercetin‐3‐glucoside (Q3 G), a flavonoid monomer compound of AP with anticervical cancer activities, induced cell cycle arrest in the S phase in a time‐dependent manner by altering CDK2 levels. It also induced apoptosis via chromosomal DNA degradation and elevated ROS production. Moreover, Q3 G altered the expressions of apoptosis‐related proteins by activating caspase‐9/‐3, downregulating the expressions of the antiapoptotic protein B‐cell lymphoma‐2 (Bcl‐2), and upregulating the proapoptotic protein, BCL2‐associated X (BAX) (Nile et al., 2021). Apple seed extracts were shown to enhance apoptosis in endometrial cancer mice models via the TNFα/p53 pathway (Kim, 2022).

### 3.4 Apples and obesity

Obesity is a serious public health concern, especially in low‐ and middle‐income countries (Popkin et al., 2012). It is associated with increased risks of T2DM, CVDs, cancer, and premature death (Flegal et al., 2005; Seidell et al., 2001; Zhao et al., 2020). Its etiology is due to a combination of factors, with eating behaviors being the most important (Chen et al., 2020). Fruit intakes are beneficial for weight loss (Buijsse et al., 2009; He et al., 2004; Vioque et al., 2008). Clinical trials, animal studies, and cellular studies have shown that apples have the ability for protecting against obesity.

#### 3.4.1 Clinical trials

In children, obesity can lead to multiple metabolic complications, therefore, most studies are focused on childhood obesity. According to the National Health and Nutrition Examination Survey 2003–2010, consumption of any form of apples contributed to a better quality of diet and reduced the risk of obesity among children (O'Neil et al., 2015). In a related clinical trial, intake of dried apples (120 kcal) twice a day for 8 weeks elevated HDL‐C levels in overweight and obese children (aged 10–16 years) (Eisner et al., 2020). Compared to subcutaneous adipose accumulation, intra‐abdominal visceral fat accumulation is closely associated with obesity, glucose intolerance, and abnormal lipid metabolism (Kanai et al., 1990). The beneficial effects of apple polyphenols on the inhibition of visceral fat accumulation in volunteers at different BMI levels have been reported (Nagasako‐Akazome et al., 2007). Yoko et al. found that both long‐term (340 g, 12 weeks) and excess consumption of apple polyphenols suppressed visceral fat accumulation in moderately underweight to moderately obese subjects and was safe in humans (Akazome et al., 2010). It was also effective in healthy individuals with relatively high BMI (23 < BMI < 30) who consumed apple polyphenol capsules for 12 weeks (Nagasako‐Akazome et al., 2007).

#### 3.4.2 Animal and cellular studies

Highly polymeric procyanidins (PP) are nonabsorbable flavonoids. Masumoto et al. found that PP intervention attenuated weight gain and obesity‐related inflammation in C57BL/6J mice fed a high‐fat/high‐sucrose diet, mainly by lowing levels of LPS, TNFα, and IL‐6. In mice treated with PP, the expression of genes linked to the intestinal barrier (TJP1 and occludin) and liver inflammatory receptors (TLR4 and CD14) was decreased, resulting in a positive impact on the balance of intestinal microbiota (Masumoto et al., 2016). AE intake in mice attenuated HFD‐induced fat deposition, particularly visceral fat, as revealed by several clinical trials (Boqué et al., 2013). AP and apple juice supplementation, especially AP, exhibited similar outcomes in HFD‐induced obese mice models, with a bigger increase in serum HDL‐C levels and brown adipose tissues (Cho et al., 2013). In vivo and in vitro, apple polyphenols and proanthocyanins in AP inhibited pancreatic lipase activities and TG absorption (Sugiyama et al., 2007). Dietary apple polyphenols inhibited adipogenesis and reduced the weights of retroperitoneal and epididymal adipose tissues in Wister rats (Nakazato et al., 2006). Ravn et al. found that AP enhanced short‐chain fatty acid production and bile acid excretion to suppress cholesterol levels in rats (Ravn‐Haren et al., 2018). Phloretin has also been shown to reduce body weight and alleviate metabolic disorders in obese mice via the gut–microbial barrier axis (Zhang et al., 2020). In a separate study, it was found that administering phloretin increased the expression of genes responsible for fatty acid oxidation, specifically recombinant carnitine palmitoyltransferase 1a (Cpt1a) and Cpt1b, while downregulating genes associated with obesity such as MCP‐1, peroxisome proliferators‐activated receptors γ2 (PPARγ2), and Mgat‐1 (Alsanea et al., 2017). These findings suggest the potential of phloretin to reduce obesity and maintain metabolic homeostasis. After a 30‐day gavage of ACV, obese mice lost weight, consumed less food, and their blood lipid, as well as blood glucose profiles, improved (Bouderbala et al., 2016). Gut microbiota has been associated with the onset of obesity (Ménard & Smet, 2019). Intriguingly, pectin and phlorizin were shown to prevent obesity and ameliorate inflammatory responses by adjusting the disturbed intestinal flora. Moreover, they exhibited comparable effects on obesity‐induced anti‐inflammation in vitro (Liddle et al., 2020).

### 3.5 Apples in aging and cognitive impairment

The global aging population is steadily increasing (Choi et al., 2021). Older people are forgetful and at a high risk of developing dementia, which is closely associated with increased oxidative stress (Wang et al., 2019). Consumption of apples, apple products, or apple ingredients has been shown to slow aging, improve cognitive functions, and protect the nervous system. Lan et al. reported that phloridzin had an antiaging role and prolonged the lifespan of the K6001 yeast strain via the SOD gene and the Sir2 signaling pathways (Xiang et al., 2011). Apples have beneficial effects on memory impairment too. Apple polyphenols diminished memory impairment and attenuated hippocampal neuronal damage in rats. This was manifested by decreased levels of NO and MDA and increased levels of GSH in the rat hippocampus (Wang et al., 2020). Phloretin improved spatial memory in a mouse model of amnesia and upregulated the levels of antioxidative enzymes (SOD, CAT) as well as brain‐derived neurotrophic factors (BDNF) in the hippocampus (Ghumatkar et al., 2015). In addition, ACV diminishes memory impairment by protecting against oxidative stress‐induced cortico‐hippocampal neuronal degeneration (Tripathi et al., 2020; Tripathi & Mitra Mazumder, 2022). ACV expressed neuroprotective effects in Alzheimer's disease mice models by improving the levels of monoamine oxidase (MAO) as well as those of amine neurotransmitters, dopamine, serotonin, and nonadrenaline (Tripathi & Mazumder, 2022). In summary, the consumption of apples, apple products, or ingredients delays aging and alleviates cognitive deficits via antioxidant effects.

### 3.6 Apples and alopecia

Alopecia (baldness) may lead to psychological distress (Hawkshaw et al., 2018) and is a common problem in cosmetics and primary health practice (Semalty et al., 2011). Hair loss individuals often inquire if nutritional supplements can help restore hair growth or arrest disease progression (Guo & Katta, 2017). Extractions called AMS, derived from Anurka apples, are known for their high levels of oligomeric procyanidin, which exhibited noticeable effects on alopecia in a clinical trial. After taking AMS daily for 2 months, 250 patients showed increased hair growth and density by promoting keratin biosynthesis. These effects were attributed to apple procyanidin B2 in AMS (Tenore et al., 2018). Another randomized controlled clinical trial validated the effects of procyanidin B2 in promoting hair regrowth among balding volunteers (30 males) receiving a 1% procyanidin B2 dose for 4 months (Takahashi et al., 2001). In mice, procyanidin B‐2 exhibited similar effects, and toxicology studies have confirmed its safety on human skin (Tenore et al., 2018). Thus, proanthocyanidins are promising therapeutic options for hair loss. Hair growth effects of procyanidin oligomers are also associated with promoting hair epithelial cells by activating MEK and preventing TGF‐β1‐ or TGF‐β2‐induced apoptosis (Kamimura et al., 2006).

### 3.7 Other health benefits of apples

Apples are rich in fiber and polyphenols interfere with plaque formation and the production of acidogenicity by oral bacteria (Touger‐Decker & van Loveren, 2003). A previous study investigated whether chewing apples mechanically removed plaque or inhibited the viability of bacteria in saliva. In the clinical trial, 20 healthy adults with good oral health status were randomly asked to brush their teeth or eat an apple. After 2 weeks, the experiment was repeated in reverse order. It was established that chewing an apple did not remove dental plaque, but it did immediately reduce the viability of salivary bacteria, similar to brushing your teeth (Rubido et al., 2018). High‐dose niacin significantly improved the levels of all major lipoproteins. However, its use was greatly limited by secondary bothersome flushing of the skin. Clinical and anecdotal evidence suggested that apple pectin has the potential to be an alternative to aspirin for preventing niacin‐induced flushing (Moriarty et al., 2013). Apple pectin solution was also found to inhibit burn wound inflammation and accelerated epithelialization in II–IIIA degree burns (Lazareva et al., 2002). Apple active ingredients or apple products also have positive effects in alleviating UV‐induced skin pigmentation (Shoji et al., 2020), persistent allergic rhinitis (Enomoto et al., 2006), cedar pollinosis (Kishi et al., 2005), and enhanced resistance training‐induced skeletal muscle hypertrophy (Gonzalez et al., 2020; Joy et al., 2015; Joy, Vogel, Moon, Falcone, Mosman, & Kim, 2016; Joy, Vogel, Moon, Falcone, Mosman, Pietrzkowski, et al., 2016).

## 4 POTENTIAL HARMFUL EFFECTS OF APPLES ON THE HUMAN BODY

There are concerns with regard to the side effects of consuming apples or apple products. In healthy subjects, apple juice improved plasma antioxidant activities by elevating plasma uric acid levels, which were dependent on the intake of fructose from apples (Godycki‐Cwirko et al., 2010). Some people, such as people with Gout, cannot tolerate elevated uric acid levels. The mechanism involved in fructose‐induced increase in plasma uric acid levels has not been fully established, however, two possible mechanisms have been hypothesized. First, it is believed that fructose is metabolized by the liver, consuming large amounts of adenosine triphosphate, thereby increasing uric acid levels (Brown et al., 2011). On the other hand, fructose indirectly reduces plasma uric acid levels by leading to insulin resistance (Dhar et al., 2011). Even though uric acid is an antioxidant, it may increase the risk of various diseases. As a consequence of long‐term high uric acid levels, tophi accumulate in the kidney, causing renal tissue necrosis or uremia. Furthermore, serum uric acid levels are closely associated with hypertension, dyslipidemia, obesity, impaired glucose metabolism, and CVD (Rafiullah et al., 2020; Wu et al., 2016). Therefore, people with elevated plasma uric acid levels should less frequently eat apples. Alternatively, eat the apple in its whole form as it provides a high amount of dietary fiber, which increases satiety. A randomized controlled study published in 2013 reported that serum LDL‐C levels decreased after the consumption of whole apples, pomace, and cloudy juices (Ravn‐Haren et al., 2013). LDL‐C concentrations increased by 6.9% after drinking clear apple juice, compared to whole apples and pomace (Ravn‐Haren et al., 2013). The reduction in fiber levels was due to the lack of pectin and other components of cell walls in clear apple juice. Thus, clear apple juice may not be a suitable substitute for whole apples.

Excess consumption of fruits is harmful to health. Excess consumption of fruits in the second trimester is associated with increased incidences of GDM. However, GDM incidences have also been associated with moderate‐to‐high glycemic index fruits, tropical fruits, and citrus fruits, but not apple products (Huang et al., 2017). Another study found that there is no correlation between prepregnancy fruit consumption and GDM incidences (Chen et al., 2012). Akazome et al. evaluated the safety of excess intake of apple polyphenol beverages. There were no adverse effects in either long‐term (12 weeks) or excess (three times that of conventional beverages) intake of apple polyphenol beverages (Akazome et al., 2010). Studies have also found that apple consumption prevents diabetes and reduces the risk of T2DM (Mursu et al., 2014; Song et al., 2005).

## 5 OPPORTUNITIES AND CHALLENGES FOR THE UTILITY OF APPLES

Microbial growth, tissue softening, and water and nutrient loss are the primary issues that fresh apples face during storage. Currently, there are several common technologies used for preserving apples, including low‐temperature preservation, modified atmosphere preservation, short‐wave ultraviolet preservation, ozone preservation, and microbial preservation (Oyenihi et al., 2022). According to recent studies, treating apples with hot water and electrolyzed water has been found to extend their storage time (Nyamende et al., 2021). Both treatments aid in removing chemical residues from the fruit and have a strong bactericidal effect. Additionally, electrolytic water acts as a scavenger of free radicals (Kim & Hung, 2014). Ahmed et al. developed new films using Ditriterpenoids and Secomeliacins isolated from *Melia azedarach* (Dharek) and *Azadirachta indica* plants. These films are a promising technique for maintaining the quality of fruits and preserving their bioactive compounds (Ahmed et al., 2022). In conclusion, the demand for apple storage and preservation is increasing, which necessitates the optimization and improvement of existing preservation technology. Additionally, further molecular mechanism studies on the causes of apple aging, browning, and rotting may provide novel insights for enhancing apple storage and preservation technology.

To tackle the challenges related to storage and transportation, apples are often processed into juice drinks. The process typically involves washing, grinding, and pressing apples to extract the juice (Gökmen et al., 2003), which can be either clear or cloudy depending on how solid particles and pectin are removed. Cloudy apple juice is made clear by removing suspended solids and pectin. Unfortunately, this process leads to a loss of various beneficial components, including phenolic compounds (Gökmen et al., 2003; Onsekizoglu et al., 2010) and vitamin C (Tchuenchieu et al., 2018). The 2015–2020 Dietary Guidelines for Americans acknowledged that 100% fruit juice can fulfill a person's daily fruit requirements (Byrd‐Bredbenner et al., 2017). Furthermore, a study demonstrated that consuming 100% juice is linked to improved diet quality and increased nutritional intake (Agarwal et al., 2019). Although some studies suggested that apple juice may offer health benefits, other research showed that the juicing process can result in significant nutrient loss (Ravn‐Haren et al., 2013; Soler et al., 2009). Therefore, it is important to note that apple juice should not be considered a substitute for whole apples in a healthy diet. According to a previous study, clear apple juice was found to have a significant negative effect on blood lipids, while cloudy apple juice had a positive impact on lowering cholesterol levels (Ravn‐Haren et al., 2013). As a result, it is recommended to choose cloudy apple juice over clear apple juice or whole apples.

The health benefits and disease prevention properties of apples and their derivatives, including juice, puree, vinegar, and extracts, have become increasingly well known. The specific characteristics of these apple products vary depending on the processing methods used. ACV was found to possess strong antioxidant properties, as well as the ability to lower lipids, regulate blood pressure (Qiu et al., 2010), and exhibit antibacterial activities (Bakir et al., 2016). ACV, an acidic solution produced by fermenting apples, is commonly used as a food flavoring and preservative (Khezri et al., 2018). Research showed that ACV may have a positive impact on various health conditions such as CVD, diabetes, memory disorders, obesity, and aging. It is considered a functional food and adjunctive treatment for metabolic abnormalities. However, further studies are needed to fully understand the potential benefits of ACV on glycemic markers and lipid profiles. Apple puree is used to produce apple sauce, pulp, cloudy juice, baby food, etc. Flavanol‐rich apple puree increased plasma NO metabolites and is beneficial for CVD (Gasper et al., 2014). Further research is necessary to fully understand the potential benefits of apple puree. In the past, AP was considered a waste by‐product of apple processing, prone to serious pollution of the natural environment and public health hazards (Skinner et al., 2018). The recent article highlighted the potential use of apple seeds in various industries such as food, pharmaceutical, and nutraceutical, emphasizing the versatility of apple ingredients (Kumar et al., 2022). The commercial value of AP has been gradually recognized by researchers. And the active compounds and raw materials derived from it can be utilized as antioxidants, preservatives, wood protectors, biopolymers, and more (Gołębiewska et al., 2022). AP is a valuable source of polyphenols and soluble fiber, which have been shown to have positive effects on reducing lipid levels, providing antioxidant benefits, and promoting neuroprotection (Alawadi et al., 2023). Further investigation is warranted to explore the potential of AP in improving host gut health and gut microbiota (Jackson et al., 2022). The popularity of dried apples is on the rise due to the fact that dehydration minimizes the risk of food spoilage. As a complementary food, dried apples have been shown to alleviate symptoms in both obese and diabetic individuals. Additionally, dried apples show potential as a substitute for fresh apples.

In addition to apple products, the active ingredients of apples also show great potential in research. Apples are a rich source of bioactive components, with polyphenols being the main contributors to the health benefits they offer. A recent review conducted by Ananya et al. provided a detailed summary of the antioxidant, cytotoxic, anti‐inflammatory, antihypertensive, and antidiabetic effects of polyphenols (Rana et al., 2022). Our review also confirms that apple polyphenols have beneficial effects on various health conditions including CVD, cancer, diabetes, cognitive dysfunction, obesity, aging, and even hair loss. Therefore, apple polyphenols are the main drivers behind the multiple health benefits that apples offer. The triterpenes in apples exert good anti‐inflammatory effects. While pectin has cholesterol‐lowering, hypoglycemic, and antioxidant properties, different pectin composites or blends can be produced as membranes, sponges, hydrogels, or 3D‐printing matrices for tissue regeneration applications (Bostancı et al., 2022). However, it is important to note that further clinical trials are necessary to validate and ensure the safety of these applications.

## 6 SUMMARY AND CONCLUSIONS

**FIGURE 2:**
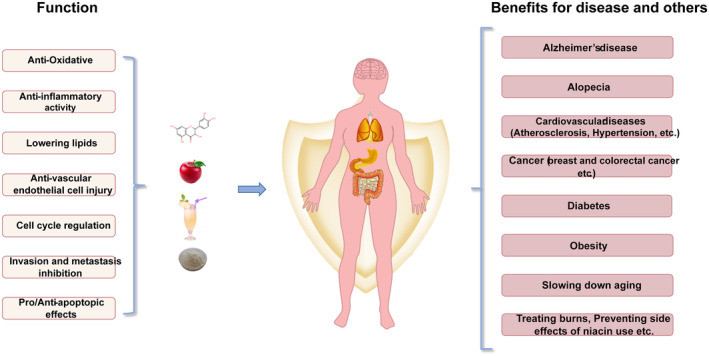
The effects of apples and their products on people's health.

**FIGURE 3:**
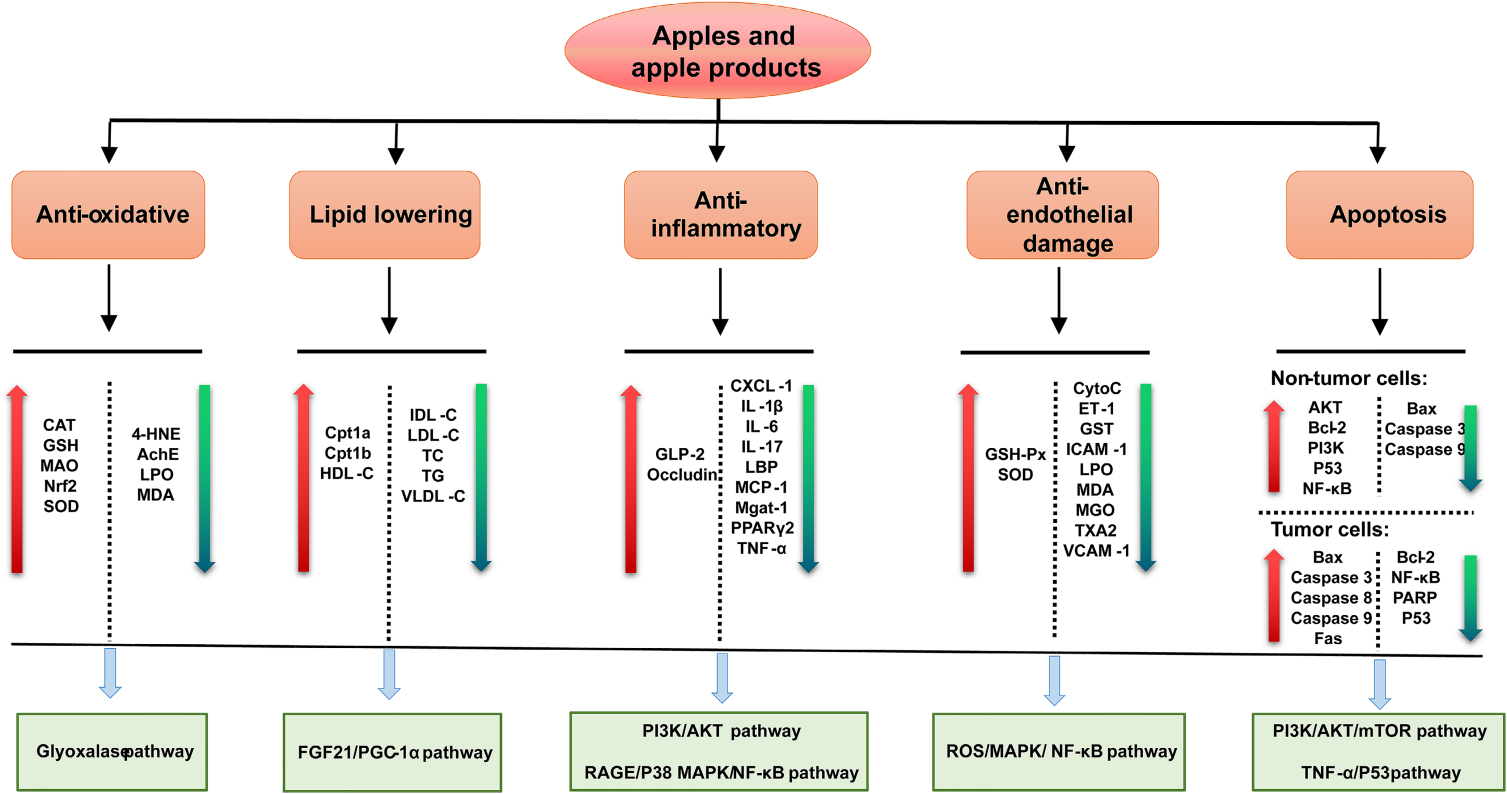
The key signaling pathways and targets of apples and their products prevent and against diseases. The orange boxes indicate the mechanisms involved, and just below are their respective up‐ or downregulated targets (red, upregulated; blue, downregulated). The green boxes show the specific pathways involved in each mechanism.

**FIGURE 4:**
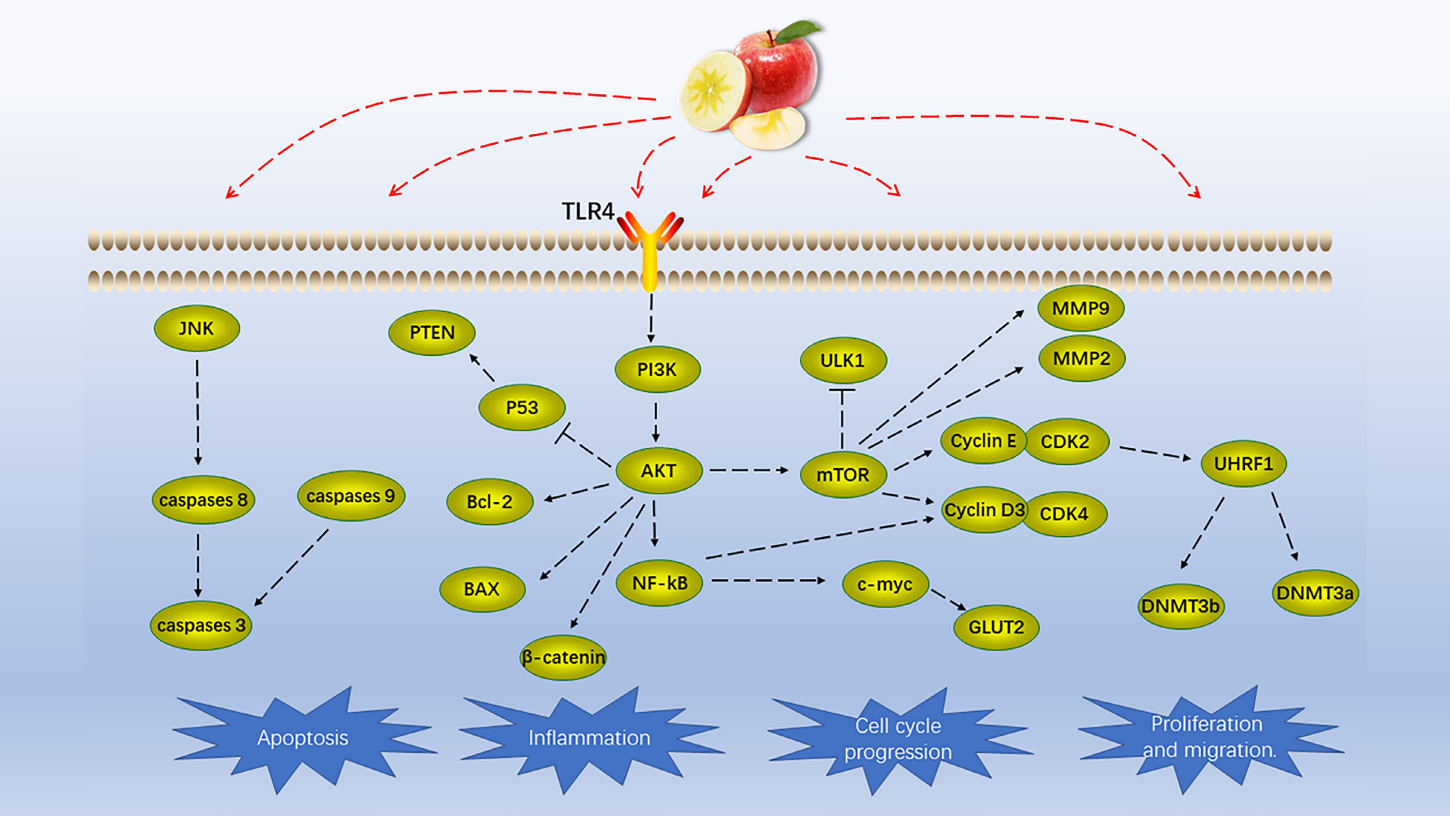
Anticancer mechanism of apples and their products.

Apples, apple components, and apple products have multiple health benefits (Figure 2). Apple products have protective effects against CVD and cancer (see Supplementary Table S1 and Table S2 for further details), as well as cognitive functions, and promote hair growth, healing of burn wounds, improve the oral environment, prevent niacin‐induced skin flushing, promote the relief of UV‐induced skin pigmentation, and improve the symptoms of atopic dermatitis as well as cedar hay fever among others. These effects are associated with various mechanisms, such as antioxidation, protecting vascular endothelium, reducing blood lipids, anti‐inflammatory, and antiapoptotic (Figure 3). At the same time, we also summarized the anticancer mechanism of apples (Figure 4).

The glyoxalase pathway is a highly conserved antioxidant defense system consisting of protein glyoxalase I, glyoxalase II, and catalysis‐reduced GSH (Sousa Silva et al., 2013). This pathway helps neutralize highly reactive and oxidizable dicarbonyl molecules, thereby maintaining MGO at a nontoxic level for cells (Allaman et al., 2015). FGF21, which is mainly secreted by the liver, positively regulates the peroxisome proliferator‐activated receptor gamma coactivator 1α (PGC‐1α) through a feed‐forward autocrine/paracrine loop in WAT. The intervention of apple polyphenols increased the antioxidant capacity of cells, while trapping harmful compounds (MGO) to combat oxidative stress (Cianfruglia et al., 2020). And the research has identified the promotion of white fat browning by targeting the FGF21–PGC‐1α axis as an attractive treatment for obesity and related metabolic disorders. Dietary administration of apple polyphenols induced beige adipocyte development in white inguinal fat of obese mice and upregulated FGF21‐PGC‐1α expression to reduce adipose tissue mass. The PI3K/Akt signaling pathway regulates the process of apoptosis and inflammatory response. Quercetin, a potential dietary flavonoid, can modulate downstream apoptosis and inflammation‐related indicators partly through PI3K/Akt to combat atherosclerosis (Lu et al., 2017). Xu et al. demonstrated that APs have the potential to prevent MAPK/NF‐κB activation induced by ox‐LDL, thereby reducing endothelial inflammation and inhibiting early atherogenic lesions. These findings confirm the beneficial effects of APs as nutritional supplements in reducing atherosclerosis (Xu et al., 2015). The interaction between AGEs and RAGE leads to the phosphorylation of MAPKs, which in turn induces aberrant activation of NF‐κB. This activation induced the expression of RAGE, resulting in a closed loop of the inflammatory response mentioned above. This loop can have serious consequences for the organism (Zhou, Xu, et al., 2019). Therefore, it is suggested that reducing RAGE levels may be an important means to prevent inflammation induced by AGEs. Phloretin was found to inhibit the RAGE/p38 MAPK/NF‐κB signaling pathway to exert its anti‐inflammatory effects (Zhou, Gong, & Wang, 2019).

The mTOR/AKT pathway functions centrally in cell proliferation and growth and is frequently overactivated in breast cancer (Nunnery & Mayer, 2020). Meanwhile, the mTOR/AKT cascade inhibits the initiation of autophagy by suppressing ULK1, which may form protein complexes and play a key role in autophagy initiation (Ramachandran et al., 2021). One study found that apple dihydrochalcone phloretin has the ability to inhibit the growth of breast cancer cells. This is achieved by suppressing cytoprotective autophagy through the downregulation of mTOR/ULK1 signaling (Chen et al., 2021). NF‐κB pathway is involved in the regulation of the tumor cell cycle and apoptosis (Shin et al., 2017), and TLR‐4 is an upstream molecule for NF‐κB (Qi et al., 2021). Apple oligogalactan combined with 5‐FU treatment effectively suppressed NF‐κB activation by inhibiting the degradation and phosphorylation of IκB to block the translocation of NF‐κB (p65) and enhanced the tumor suppressive effect of 5‐FU. This was achieved by affecting the TLR‐4/NF‐κB pathway (Li et al., 2017). The TNFα/p53 pathway was activated to induce apoptosis, and the intervention of apple seed extract resulted in a significant increase in apoptosis in endometrial cancer cells (Kim, 2022). This further highlights the advantageous impact of apple components on cancer. The PI3K/AKT/mTOR pathway plays a fundamental role in cancer cell proliferation, survival, and metabolism, and is one of the most commonly disrupted pathways in malignancy (Lindsley, 2010). Ruthenium–phloretin complex regulated the PI3K/Akt/mTOR pathway with MMP9 to inhibit tumor invasion and arrest breast cancer progression (Roy et al., 2022). In recent years, the PI3K/AKT/mTOR pathway has become a promising target for treating malignancies. As a result, there has been a surge in research on inhibitors targeting this pathway. There are currently available inhibitors for PI3K, AKT, mTOR, and Dual PI3K/mTOR, however, most of them produce multiple adverse effects and are prone to drug resistance (Peng et al., 2022). Therefore, plant‐based active ingredients may be a more rational treatment option that requires further in‐depth research.

In conclusion, the effects of apples and apple derivatives on disease risk reduction are both challenging and encouraging. The combined phytochemical and nutrient profiles in apples suggested their potential to be powerful for the prevention of several chronic conditions in humans. Studies are aimed at delineating multiple mechanisms by which apples and apple products might be protective.

## AUTHOR CONTRIBUTIONS

**Yue Zhang:** Conceptualization (supporting); project administration (equal); visualization (equal); writing – original draft (lead). **Miao Zeng:** Investigation (supporting); project administration (equal); supervision (lead); writing – original draft (equal). **Xiaolu Zhang:** Investigation (equal); project administration (equal); visualization (equal); writing – original draft (equal). **Qun Yu:** Methodology (equal); visualization (equal); writing – review and editing (equal). **Wenyun Zeng:** Formal analysis (equal); methodology (equal); validation (equal). **Bin Yu:** Funding acquisition (equal); investigation (equal); writing – review and editing (equal). **Jiali Gan:** Data curation (lead); resources (equal); writing – review and editing (equal). **Shiwu Zhang:** Project administration (equal); visualization (equal); writing – review and editing (equal). **Xijuan Jiang:** Funding acquisition (equal); project administration (equal); writing – review and editing (lead).

## CONFLICT OF INTEREST STATEMENT

The authors declare that there are no conflicts of interest.

## ETHICS STATEMENT

This study did not involve any human or animal testing.

## INFORMED CONSENT

This review article did not involve study participants, and informed consent was not required.

## Supporting information


Table S1–S2.

## Data Availability

Data sharing is not applicable to this article as no new data were created or analyzed in this article.
