# Supplementary material for: Does an apple a day keep away diseases? Evidence and mechanism of action
Source: Food Sci Nutr. 2023 Jun 20;11(9):4926–47. doi: 10.1002/fsn3.3487 (PMC10494637; doi:10.1002/fsn3.3487)
Supplement: Supplementary file 1 — Table S1–S2. [file FSN3-11-4926-s001.docx]

Table S1 Summary of all clinical trials in the present review

| Authors | Purpose | Subjects | Intervention | Comparison | Outcomes |
| --- | --- | --- | --- | --- | --- |
| Hodgson JM 2016 | To examine the association of apple intake with all-cause and disease-specific mortality over 15 years in a cohort of women. | 1,456 women aged over 70 years | Apple and other fruits intake | Cardiovascular and cancer mortality | Higher apple intake was associated with a lower risk for cancer mortality. |
| Knekt P 2002 | To examine the association between flavonoid intake and the risk of several chronic diseases. | 10,054 men and women | Dietary flavonoids | Incidence of chronic diseases, such as lung cancer, diabetes, IHD mortality, total mortality, etc. | The risk of some chronic diseases may be lower at higher dietary flavonoid intakes. |
| McCullough ML 2012 | To examine the association between flavonoid intake and CVD mortality among participants in a large, prospective US cohort. | 38,180 men and 60,289 women | Dietary flavonoids | Risk of fatal CVD, stroke, ischemic heart disease | Five flavonoid classes (anthocyanidins, flavan-3-ols, flavones, flavonols, and proanthocyanidins) were associated with a lower risk of fatal CVD. |
| Bondonno CP 2011 | To investigate the independent and additive effect of flavonoid-rich apple and nitrate-rich spinach on NO status, endothelial function, and BP. | 30 healthy volunteers (6 males, 24 females) | Control, apple, spinach, and apple+spinach. | NO status, endothelial function, and BP | Flavonoid-rich apple and nitrate-rich spinach can independently augment NO status, enhance endothelial function, and lower BP acutely. |
| Yuan LH 2011 | To investigate the influence of apple and grape juice consumption on body antioxidant status. | 26 healthy young subjects (13 male and 13 female) | Fruit juice twice a day (300 mL apple juice at lunch and 300 mL grape juice at dinner) | Plasma T-AOC, MDA, carbonyl， erythrocyte antioxidant enzymes activities, Urinary 8OHdG content | Increased the plasma T-AOC and decreased the concentration of MAD, erythrocyte GSH-Px, and CAT activities were enhanced. |
| Soriano-Maldonado A 2014 | To investigate the effect of the consumption of two cloudy apple juices with different polyphenol and vitamin C contents on antioxidant status, cardiometabolic, and inflammation markers. | 20 healthy volunteers (12 women and 8 men) | Volunteers randomly consumed two glasses of either a vitamin C-rich apple juice or a polyphenol-rich juice | Markers of antioxidant status, glucose metabolism, lipid profile, and inflammation | During the vitamin C-rich apple juice period, plasma antioxidant activity increased, while ICAM-1 and TC decrease.  During the polyphenol-rich juice period, plasma insulin and HOMA increased, and total glutathione decreased. |
| Ko SH 2005 | To test the hypothesis that the consumption of fruit juices may improve antioxidant status in human plasma. | 10 healthy men | Study subjects were fed 150 mL of fruit juice (pear, apple, orange, grape, peach, plum, kiwi, melon, and watermelon) | Measurement of antioxidant activity in human plasma | Within 30 minutes after consumption, orange, melon, grape, peach, plum, apple, and kiwi juices already effectively suppressed ROS generation. |
| Godycki-Cwirko M 2010 | To determine whether (1) rapid consumption of 1 L of apple juice increases blood antioxidant capacity, and (2) apple polyphenols or fructose-induced elevation of plasma uric acid contributes to increasing blood antioxidant activity. | 12 healthy nonsmoking subjects | The volunteers drank either 1 L of apple juice without polyphenols, 1 L of water, or 1 L of cloudy apple juice | DPPH and FRAP radical-scavenging activity, serum uric acid | Rapid consumption of apple juice increased plasma antioxidant activity (FRAP and DPPH) in healthy subjects. |
| Saarenhovi M 2017 | To test that an orally ingested apple polyphenol extracts rich in epicatechin and flavan-3-ol oligomers improve endothelium-dependent brachial artery FMD and endothelium-independent NMD. | 60 healthy subjects (26 men, 34 women) with borderline hypertension (BP 130-139/85-89 mmHg) or unmedicated mild hypertension (BP 140-165/90-95 mmHg) | Placebo or the apple polyphenol extract to provide a daily dose of 100 mg epicatechin | FMD and NMD | A significant acute improvement in maximum FMD% with AE administration. |
| Bondonno NP 2018 | To determine if acute and/or chronic ingestion of flavonoid-rich apples improves endothelial function, BP, and arterial stiffness in individuals at risk for CVD. | 30 volunteers were included in the study if they had at least one of the following: elevated BP (120 mmHg<systolic BP<160mmHg), moderately elevated blood sugar concentrations (5.6 mmol/L< glucose <6.5  mmol/L), raised fasting cholesterol (5 mmol/L< TC <8 mmol/L) or central obesity (waist circumference; men >94 cm; women >80 cm) | Apple with skin is compared to intake of apple flesh only | Endothelial function is assessed using FMD of the brachial artery, while the main secondary outcomes are 24 h ambulatory BP and arterial stiffness | Compared to apple flesh only control, the apple with skin resulted in a significant increase in FMD acutely. |
| Chai SC 2012 | To evaluate the effect of dried apple vs dried plum consumption in reducing CVD risk factors. | 160 postmenopausal women | Dried apple (75 g/day) or dried plum | Serum TC, LDL-C， lipid hydroperoxide, and CRP | These serum values were further decreased to 13% and 24%, respectively, after 6 months. And both dried fruits were able to lower serum levels of lipid hydroperoxide and CRP. |
| Ravn-Haren G 2012 | To evaluate the influence of fruit may cause protection by affecting common risk factors of CVD. | 23 healthy volunteers | Whole apples, AP, clear and cloudy apple juices | TC and LDL-C， HDL-C, TG, weight, waist-to-hip ratio, BP, inflammation, the composition of the gut microbiota, or markers of glucose metabolism | A lower serum LDL-C was observed after whole apple (6.7%), pomace (7.9%), and cloudy juice (2.2%) intake. |
| Koutsos A 2020 | To determine the effects of apple consumption on circulating lipids, vascular function, and other CVD risk markers. | 40 healthy mildly hypercholesterolemic volunteers (23 women, 17 men) | 2 apples/d (Renetta Canada) or sugar- and energy-matched apple control beverage | Serum lipids, glucose, insulin, bile acids, endothelial and inflammation biomarkers， and arterial stiffness | Whole apple consumption decreased serum TC and LDL-C, TG, and intercellular CADM1, and increased serum uric acid.  The response to endothelium-dependent microvascular vasodilation was greater after the apples than after the control beverage. |
| Tenore GC 2017 | To determine the influence of daily consumption of Annurca apples on cholesterol levels. | 250 mildly hypercholesterolemic healthy subjects | Four groups were administered one apple per day among the following: Red Delicious, Granny Smith, Fuji, and Golden Delicious, the fifth group was asked to consume two Annurca apples per day | Plasma TC, LDL-C, and HDL-C levels | Annurca led to the most significant outcome, allowing a reduction in TC and LDL-C levels by 8.3% and 14.5%, respectively, and an increase in HDL-C levels by 15.2%. |
| Tenore GC 2017 | To determine the healthy balance of plasma cholesterol by a novel Annurca apple-based nutraceutical formulation. | Patients were eligible for enrolment if they had the following values of serum cholesterol parameters at baseline: TC, 200–260 mg/dL; HDL-C, 30–45 mg/dL; and LDL-C, 189–206 mg/dL | Capsules of AMS or identically appearing capsules containing only maltodextrin | Plasma TC, HDL-C, and LDL-C levels | Two AMS capsules a day, after one month, lowered LDL-C equivalent to 40 mg of simvastatin or 10 mg of atorvastatin. Meanwhile, AMS had a significant effect on HDL (+49.2%). |
| Tenore GC 2019 | To determine the effects of lactofermented Annurca apple puree on HDL-C and TMAO levels in subjects. | 90 individuals with CVD risk factors | Lactofermented Annurca apple puree， unfermented apple puree, or probiotic alone | Plasma lipid profile and TMAO levels， HDL-C | Subjects tested with lactofermented Annurca apple puree experienced changes in the following serum parameters: HDL-C, +61.8%; and TMAO levels, -63.1%. |
| D'Assante R 2021 | To evaluate the effect of Melasterol in eighty-seven patients with acquired hyper-cholesterolemia. | 87 patients with acquired hyper-cholesterolemia | Tablet of Melasterol | Plasma TC, HDL, LDL, TGs levels, body weight, and glycemia | Six months of treatment resulted in a 19.2% reduction in TC, a 19.8% reduction in LDL, a 23% reduction in TG, and significant reductions in blood glucose and BMI. |
| Liddle DM 2021 | To determine the effects of apple consumption on fasting plasma biomarkers of inflammation, endotoxemia, carbohydrate and lipid metabolism, and PBMC-secreted cytokines in individuals. | 46 overweight and obese participants | 3 whole Gala apples as part of their habitual diet or avoided apples | Fasting plasma biomarkers of inflammation, endotoxemia, carbohydrate and lipid metabolism, and PBMC-secreted cytokines | Apples decreased fasting plasma CRP by 17.0%, IL-6 by 12.4%, and LBP by 20.7%.  Apples decreased PBMC-secreted IL-6 by 28.3% and IL-17 by 11.0%.  Apples also increased plasma T-AOC by 9.6%. |
| Chun OK 2008 | To test the associations between dietary flavonoid intake and serum CRP concentrations among U.S. adults. | 8,335 adults, excluding pregnant and/or breastfeeding women | Dietary flavonoids | Serum CRP concentrations | Intakes of apples and vegetables were inversely associated with serum CRP concentrations. |
| Schulze C 2014 | To demonstrate that an extract and individual polyphenols from apple diminish SGLT1 mediated glucose uptake. | 10 healthy lean men | The capsuled AE | OGGT， venous blood glucose,e and plasma insulin levels | An OGGT performed in volunteers with prior administration of the AE reduced venous blood glucose and plasma insulin levels. |
| Sansone K 2018 | To assess the potential of dried apple to regulate acute blood glucose, insulin, satiety, and total plasma antioxidant levels, and to explore the effect of dried apple on cognitive responses. | 21 healthy, normal-weight subjects | Dried apple or muffins | Blood glucose, insulin, and antioxidant concentrations were measured at various postprandial time points, and satiety was assessed. | Consumption of the dried apples produced significantly lower glucose concentrations at 30-, 45-, 60-, and 120-min time points, and significantly lower insulin concentrations at a 15-min time point than the muffins. |
| Makarova E 2015 | To prepare a low-sugar, fiber- and phlorizin-enriched powder from unripe apples and to gain insight regarding its anti-hyperglycemic activity in healthy volunteers. | 6 healthy volunteers | 50g OGTT or an OGTT with the addition of 25 g of the apple preparation | OGTT | Acute ingestion of the apple preparation reduced the postprandial glucose response and by increasing urinary glucose excretion. |
| Shoji T 2017 | To evaluate the effects of chronic apple polyphenols administration on glucose tolerance in high-normal and borderline human subjects. | 65 subjects with an FPG level of 100-125mg/dL | Tablets containing apple polyphenols or placebo tablets | Insulin resistance was assessed using a 75g OGTT | Chronic administration of apple polyphenols significantly reduced the increase in glucose at a 30-min OGTT value, compared to the placebo regimen.  In a subgroup of the high-normal and borderline subjects, OGTT 30-min glucose value in the apple polyphenols group was significantly lower than that of the placebo group. |
| Johnston CS 2010 | To investigate the vinegar dosage, timing, and application for reducing PPG. | 38 adults with type 2 diabetes or without diabetes | Different vinegar dosage (10 vs. 20 g), timing (during mealtime vs. 5 h before meal), and application (acetic acid as vinegar vs. neutralized salt) | PPG | Consuming two teaspoons of vinegar at mealtime can effectively reduce PPG. |
| Gheflati A 2019 | To assess the health effects of apple vinegar in patients with diabetes and dyslipidemia. | 70 participants with type 2 diabetes and hyperlipidemia | Apple vinegar | FBS, homeostasis model assessment for insulin resistance, homeostasis model assessment for B-cell function, quantitative insulin sensitivity checks index, insulin, MDA, DPPH, homocysteine, systolic BP, and diastolic BP | The intervention with apple vinegar could significantly improve FBS and DPPH within the intervention group. Glycemic indices containing insulin, HOMA-IR, HOMA-B, and QUICKI decrease significantly in both groups. |
| Laue C 2019 | To investigate the effect on postprandial glycemic and venous serum insulin response of an apple drink following the conversion of its glucose to gluconate. | 30 male adults with IFG | A drink of 500 mL: 1. Verum: Apple juice treated with invertase, glucose oxidase/ CAT (glucose 0.05 g; gluconate 18.2 g); 2. Control: Untreated apple juice (free glucose 8.5 g; bound glucose 6.7 g) | Postprandial fingertip capillary blood glucose and venous serum insulin were measured at various time points, and gastrointestinal symptoms, stool consistency, and satiety were also assessed | By enzymatic treatment of apple juice, its sugar content could be reduced by 21% and postprandial glycemic and venous serum insulin response by 68 and 47%, respectively resulting in a reduction of glycemic load by 74.6%. |
| Li H 2021 | To determine the association between mid-pregnancy consumption of fruit, vegetable, and fruit juice and the risk of GDM. | 2987 pregnant women | Fruit, vegetable, and fruit juice | The incidence of GDM | The quantity of apple, orange and vegetables were negatively associated with the incidence of GDM. |
| Askari F 2014 | To examine the association between intake of fruits and vegetables and PCa risk in Iran. | 50 patients with PCa and 100 controls | Fruits and vegetable intake | The incidence of PCa | The results of this study showed that fruit and vegetable intake was inversely associated with PCa risk. |
| Freedman ND 2008 | To determine whether fruit and vegetable intake can reduce the incidence of head and neck cancer. | 490,802 United States participants | Fruits and vegetable intake | The incidence of head and neck cancer | Total fruit and vegetable intake were inversely associated with head and neck cancer risk. |
| Eisner A 2020 | To examine whether consuming the dried apple is a practical solution for weight loss and improves body composition and metabolic markers. | 38 overweight or obese children | Dried apple or a control snack (muffin) | Weight, serum concentrations of lipids, glucose, insulin, proinsulin, total adiponectin, CRP and so on | HDL-C concentration increased after the 8-week treatment within the apple group. |
| Nagasako-Akazome Y 2007 | To evaluate the efficacy of a 12-week intake of polyphenols extracted from apples and hop bract. | 71 moderately obese male and female subjects | Apple polyphenol-containing capsules, or hop bract polyphenol-containing capsules | TC and LDL-C levels | Taking polyphenol-containing capsules for 12 weeks significantly reduced TC and LDL-C levels. |
| Akazome Y 2010 | To evaluate the safety of an excessive intake and the efficacy of a long-term intake of polyphenols derived from apples for moderately underweight to moderately obese subjects. | Moderately obese subjects (long-term intake: 94 subjects; excessive intake: 30 subjects) | A group that drank beverages with apple polyphenols and a group that drank beverages without apple polyphenols | VFA, blood examinations, or physical examinations | Compared with baseline, subjects in the apple group of the long-term ingestion trial had a significant decrease in VFA.  Compared with the placebo group, subjects in the apple group had significantly lower VFA. |
| Tenore GC 2018 | To prove the impact of apple procyanidin B2 on keratin biosynthesis in vitro, and highlights its effect as a nutraceutical on human hair growth and tropism. | Patients with hair loss | Take two capsules of AMS or AMSbzs | The increase in the hair number/cm^2^ area of bald head skin and the increase in the weight and keratin content of hair samples collected | After 2 months, hair growth, weight, and keratin content increased by 118.3%, 37.3%, and 35.7%, respectively. |
| Takahashi T 2001 | To investigate the effects of topical application of procyanidin B-2 on the scalp and hair. | 30 volunteer subjects were chosen by pre-examination under the criteria that they showed male pattern baldness on the scalp. | One group was treated with a 1% procyanidin B-2 agent, and the other group was treated with placebo control. | Hair diameter， hair density， clinical diagnosis, any effects on scalp condition, and any effects on baldness | In the procyanidin B-2 group, there was a mean increase in hair diameter of 78.9%.  The increase in the proportion of hairs over 40 microm in diameter and the total number of hairs in the designated scalp area (0.25 cm^2^) measured after 4 months of procyanidin B-2 treatment was significantly greater than in the placebo control group. |
| Kamimura A 2001 | To investigate the effects of 1% procyanidin B-2 tonic on human hair growth after sequential use for 6 months. | From 50 applicants, 30 volunteer subjects were chosen by pre-examination under the criteria that they showed male pattern baldness on the scalp. | One group was treated with a 1 % procyanidin B-2 agent, and the other group was treated with placebo control. | Hair density and determination of terminal hair formation | Subjects in the procyanidin B-2 group had significantly greater increases in the number of total hairs in the designated scalp area and in the number of terminal hairs (defined as hairs over 60 microns in diameter) in the designated area than in the placebo control group. |
| Rubido S 2018 | To determine whether chewing an apple produced mechanical removal of dental plaque or had any effect on salivary BV. | 20 healthy adults with good oral health status | Brush their teeth or eat an apple | PI and the BV | Chewing an apple does produce an immediate reduction in salivary BV similar to that after tooth brushing. |
| Moriarty PM 2013 | That pretreatment with encapsulated apple pectin would limit the incidence, severity, time of initiation, and duration of NIF. | 100 niacin-naïve subjects | Apple pectin, apple pectin + aspirin, aspirin, or place | NIF | Significantly lowered the duration of NIF and produced nonsignificant but positive improvements in all other major flushing parameters compared with placebo. |
| Shoji T 2020 | To evaluate the effects of continuous apple polyphenol administration on facial skin conditions and pigmentation induced by ultraviolet irradiation in healthy women participants. | Healthy women participants | Tablets containing apple polyphenol (300 or 600 mg/day) or placebo | Skin pigmentation (erythema value, melanin value, L value)， water content, and trans-epidermal water loss | Continuous administration of apple polyphenol for 12 weeks significantly prevented ultraviolet irradiation-induced skin pigmentation (erythema value, melanin value, L value). |
| Enomoto T 2006 | To assess the effect of drinks containing apple polyphenols on clinical symptoms of persistent allergic rhinitis. | 33 patients with moderate or severe persistent allergic rhinitis in whom the symptoms persisted for 3 years or longer | With a low dose of apple polyphenols, or with a high dose of apple polyphenols | Changes in the clinical symptoms | Significant improvements in sneezing episodes and runny nose were observed in the polyphenol-treated group, along with a higher percentage of patients with improved turbinate swelling. |
| Kishi K 2005 | To evaluate the therapeutic effect of apple polyphenols in patients with cedar pollinosis. | 36 healthy men with cedar pollinosis who met the diagnostic criteria were enrolled as subjects | Apple polyphenols (500 mg) or placebo | Pollinosis symptoms during the study were evaluated according to the classification in the guidelines for allergic rhinitis diagnosis and treatment. | The results show that the sneezing score was significantly lower for the Ap group than with the placebo group. |
| Joy JM 2016 | To determine the effects of this supplement on athletic performance when used during 12 weeks of supervised, periodized resistance training. | 25 healthy, resistance-trained, male subjects | Subjects were supplemented once daily with either 1 serving of a proprietary blend of ancient peat and AE or an equal-volume, visually-identical placebo daily. | Strength was determined using 1RM testing in the barbell back squat, bench press, and deadlift exercises.  Peak power and peak velocity were determined during the bench press at 30% 1RM and vertical jump tests as well as a 30s Wingate test. | Supplementing with ancient peat and AE while participating in periodized resistance training may enhance performance adaptations. |
| 1RM, 1-repetition-maximum; 8OHdG, 8-hydroxydeoxyguanosine; AP, Apple Pomace; BMI, Body Mass Index; BP, Blood Pressure; BV, Bacterial Viability; CHD, Coronary Heart Disease; CRP, C-reactive protein; CVD, Cardiovascular Disease; DPPH, 2,2-diphenyl-1-picrylhydrazyl; FBS, Fasting Blood Sugar; FMD, Flow-mediated Vasodilatation; FRAP, Ferric-reducing ability of plasma; GDM, Gestational diabetes mellitus; HDL-C, High-Density Lipoprotein Cholesterol; HOMA, Homeostasismodel Assessment; ICAM-1, Intercellular cell adhesion molecule-1; IHD, Ischemic Heart Disease; IL-17, Interleukin-17; IL-6, Interleukin-6; LDL-C, Low-Density Lipoprotein Cholesterol; MDA, Malondialdehyde; NIF, Niacin-induced flushing; NMD, Nitrate-mediated vasodilatation; OGTT, Oral glucose tolerance test; PBMC, Peripheral blood mononuclear cell; Pca, Prostate cancer; PI, Plaque index; PPG, Postprandial glycemia; SGLT1, Sodium-coupled glucose transporter 1; TAMO, Trimethylamine-N-oxide; T-AOC, Total antioxidant capacity; TC, Total Cholesterol; VFA, Visceral fat area. | | | | | |

Table S2 Summary of all animal and cell studies included in the present review

|  | Part of fruit | Animal model | Cell lines/model | Dose | Mechanism |
| --- | --- | --- | --- | --- | --- |
| Bouderbala H 2016 | Apple cider vinegar | Wistar rats subjected to an HFD |  | 7mL/kg/d | The metabolic disorders caused by HFD are thwarted by taking apple cider vinegar which proves to have a satiating effect and antihyperlipidemic and hypoglycemic effects. |
| Codoñer-Franch P 2013 | Dried apple enriched with mandarin juice | TAM-induced oxidative stress in female wistar rats |  | NA | Decreased in aminotransferases, CGs and 8OHdG. |
| Halima BH 2018 | Apple cider vinegar | STZ-induced diabetic rats |  | NA | Increased the SOD, CAT, and GSH-Px levels reduced LPO levels and the indices of toxicity in the liver and kidneys. |
| Bolea G 2021 | Apple puree or phenolic extract | ApoE^-/-^ mice fed with high fat with red meat with n-6 PUFAs |  | NA | Limit 4-HNE formation during digestion and durably protect vascular function. |
| Cianfruglia L 2020 | Apple polyphenols | 50 mM glucose treated for 1 week |  | 0.4,0.8 mmol GAE/L | Increase the antioxidant capacity, on the other hand, they work directly from antioxidants and trapping for harmful compounds such as MGO. |
| Jia MF 2017 | Apple peel polyphenols | Male Kunming mice fed with 3% dietary choline |  | 300, 600 and 900 mg/kg | Reduced AST, ALT activities and MDA, CRP, and TNF-α levels, and increased the hepatic GSH-Px and SOD activities. |
| Ren DY 2016 | Apple phloretin | Mice fed with a high-choline diet |  | 200 and 400 mg/kg | Reduced TC, TGs, LDL-C, AST, ALT, ET-1, GSH-Px, MDA, and TXA2 levels, and increased SOD, GSH-Px serum PGI2, HDL-C, and NO levels. |
| Nishigaki I 2010 | AE |  | HUVEC exposed to GFBS/ FeCl_3_ | 100 and 250 mg | Decreased the level of LPO, Cyto C, and GST. |
| Xu ZR 2015 | Apple polyphenols | ApoE^-/-^ mice fed with a western-type diet |  | 100 mg/kg | Suppressed the ROS/MAPK/NF-κB signaling pathway, and reduced CCL-2, ICAM-1, and VCAM-1 expression. |
| Cho KD 2013 | AP or apple juice | Obese rats fed with an HFD |  | 10% | Lower atherogenic indices and increased serum HDL-C concentration and brown adipose tissue weights. |
| Tamura Y 2020 | Apple polyphenols | C57BL/6J mice fed with HFD | Adipose-derived stem cells, an induction medium containing indomethacin, dexamethasone, 3 -isobutyl - 1 -methylxanthine, rosiglitazone and insulin | Animal:5%; Cell:0,5,15μg / mL | Activation/induction of peripheral catecholamine synthesis-FGF21-PGC-1α cascade induces the development of beige adipocytes in iWAT. |
| Tian J 2017 | Apple peel polyphenols and apple flesh polyphenols | Mice fed with a high-fat and high-fructose diet |  | 250 mg/kg | Lower BP, improved endothelial function, ameliorated lipid homeostasis, and decreased insulin resistance. |
| Sommella E 2019 | Annurca polyphenolic extract |  | human hepatocytes | 400 mg/L | Promote mitochondrial respiration, lipolysis, and fatty acids β- oxidation. Prevent citrate from being used for fat production and ultimately reduce cholesterol production and fatty acid synthesis. |
| Masumoto S 2016 | Apple procyanidins | C57 BL/6J mice fed with a high-fat/high-sucrose diet |  | 0.50% | Alleviated obesity and regulated expression of genes related to lipid metabolism, and decreased the Firmicutes/ Bacteroidetes ratio. |
| Alsanea S 2017 | Apple phloretin | HFD-induced obesity mouse model |  | 10 mg/kg | Phloretin treatment inhibited the expression of macrophage markers and pro-inflammatory genes and decreased the expression of MCP-1, Pparγ2, and Mgat-1 genes. In addition, increased expression of genes such as Cpt1a and Cpt1b, and enhanced expression of adiponectin gene in white adipose tissue. |
| Zhou Q 2019 | Apple phlorizin |  | MGO adducts induced inflammation in endothelial cells | 100 μM | Anti-inflammatory effect via RAGE/p38 MAPK/NF-κB signaling pathway. |
| Liddle DM 2020 | Apple flavonols |  | LPS- and CoCl_2_ -stimulated adipocytes | 100 μM | Reduced ROS accumulation, and NF-κB activation. |
| Lu XL 2017 | Quercetin | High fructose feeding- or LPS-induced AS mice | LPS stimulated VSMCs isolated from thoracic aortas of adult male C57 BL/6 mice | Animal:50,100mg/kg; cell:10, 20, 40 and 80mM | Reduced the atherosclerotic plaque size, and inhibited ROS production, inflammatory response, and apoptosis, which were linked with PI3K/AKT-regulated Caspase-3 and NF-κB activation. |
| Wang S 2017 | Apple polysaccharide | SD rats fed with HFD |  | 5% and 10% | Decreased plasma LBP, up-regulation of Occludin, down-regulation of TNF-a, MCP-1, CXCL-1, and IL-1β. |
| Fathy SM 2016 | Egyptian cloudy apple juice and apple peel extract | Wistar rats treated with STZ | HDL-C | 15 mL/kg cloudy apple juice and 1 g/kg apple peel extract | Decreased FPG, serum TC, TG, LDL-C, VLDL-C, and tissue MDA, NF-κB, TNF-α, IL-6, IL-8 levels coupled with the elevation of and antioxidant enzymes' activity. |
| Najafian M 2012 | Apple phloretin | An STZ-induced rat model of diabetes type 1 |  | 5, 10, 20 and 40 mg/kg | Reduced blood glucose levels and improved dyslipidemia in diabetic rats. |
| Song CL 2017 | Apple polyphenol |  | MDA-MB-231 | 0, 50, 100, 200, 400 and 800 μg/mL | Apple polyphenols inhibited the viability, cell proliferation, and migration of MDA-MB-231 by reducing UHRF1 and MMP2 protein expression while reducing DNMT3a and DNMT3b. |
| Martino E 2019 | Annurca apple polyphenol |  | MDA-MB-231 | 100,200,300,400, 500 μM | APE induces G2/M phase arrest (increased p-CDC25C, decreased cyclinA2 and cyclinB1), apoptosis (increased BAX/Bcl-2, Fas, decreased procaspases-8, -9, -3, and PARP), and autophagy (increased LC3BII/I and P62, decreased beclin-1 ). |
| Loung CY 2019 | Apple peel flavonoid fraction 4 |  | MDA-MB-231, MDA-MB-468，MCF-7 and SKBR3 | 20μg/ mL | Apple peel flavonoid fraction 4 inhibits the growth, proliferation, migration, and invasion of MDA-MB-231 cells. |
| Chen M 2020 | Apple dihydrochalcone phloretin |  | MCF7 and MDA-MB-231 | 10、50、100、200、300 μM | Suppress glucose-starvation- and chemotherapeutic-induced cytoprotective autophagy in breast cancer cell lines through downregulation of mTOR/ULK1 signaling. |
| Roy S 2022 | Apple polyphenol phloretin |  | MCF-7 and MDA-MB-231 | MCF-7:30,60,120μM; MDA-MB-231:10,30,60μM | The ruthenium-phloretin complex could modulate p53 intervention apoptosis in the breast carcinoma, initiated by the trail of intrinsic apoptosis facilitated through Bcl2 and Bax and at the same time down-regulating the PI3K/Akt/mTOR pathway coupled with MMP9 regulated tumor invasive pathways. |
| Yang KC 2009 | Apple polyphenol phloretin | SCID mouse model | Hep G2, Hep 3B, COLO 205, HT29, HL60 FHC | Animals: 10 mg/kg; cells:50–150 mM | Enhancement of paclitaxel-induced in vivo antitumor effect of phloretin in Hep G2 xenografted SCID mice. Meanwhile, apple polyphenol phloretin enhanced the anticancer effect of paclitaxel by inducing apoptosis of human hepatoma G2 cells. |
| Wu CH 2009 | Phloretin | SCID mouse model | HepG2, Hep3B, AU565, and MCF 10A | Animals: 10 mg/kg; Cells: 50–150μM | Phloretin-induced apoptosis in HepG2 cells involves inhibition of GLUT2 glucose transport mechanisms. |
| Sun Y 2018 | Modified apple polysaccharide | ICR mice were injected intraperitoneally with DMH and DSS |  | 1.25%, 2.5% and 5% | Modified apple polysaccharide effectively prevents colitis-associated colorectal cancer in ICR mice by regulating MUCI. |
| Sun Y 2020 | Apple polysaccharide | Male ICR mice were administered with AOM and DSS | Raw 264.7 cells | Animals:5%; cells: 0.5 mg/mL | Apple polysaccharide upregulates TLR-4 signaling and triggers M1 macrophage transition to prevent colon cancer. |
| Li YH 2017 | Apple oligogalactan | Colitis-associated colorectal cancer mice model | HT-29 and SW-620 cells | Animals: 20 mg/kg; cells: 0.05 or 0.1mM | Apple oligogalactan alone or in combination with 5-FU significantly suppressed proliferation, induced apoptosis and cell cycle arrest in colorectal cancer cells, and showed a growth inhibitory effect on a murine model of xenograft tumor. |
| Nile A 2021 | Quercetin-3-glucoside |  | He La cell | 10μg/ mL | Q3G arrests the cell cycle of cervical cancer cells, activates caspase-9/-3, downregulating anti-apoptosis protein B-cell lymphoma (Bcl)-2 expressions and upregulating the pro-apoptotic Bcl-2-associated X protein. |
| Kim SH 2021 | Apple seed extract | Endometrial cancer mice model |  | 100 mg (based on 30 g) in 200mL drinking water | Enhances apoptosis and the immune function-related factors in endometrial cells. By improving tissue remodeling, the extract may help to restore the endometrium. |
| Boqué N 2013 | Polyphenolic plant extracts | Rats were fed a high-fat-sucrose diet |  | Apple: 0.7g/kg | AEs prevented the increase in fat mass promoted by the high-fat-sucrose diet. And HOMA-IR index was reduced in rats fed apples. |
| Sugiyama H 2007 | Oligomeric procyanidins | Male ddY mice |  | 200 mg/kg | Apple polyphenol inhibited TGs absorption by inhibiting pancreatic lipase activity in mice and humans. |
| Nakazato K 2006 | Apple polyphenol | Wistar male rats |  | 0.50% | Significantly lower retroperitoneal and epididymal adipose tissue weights after apple polyphenol diet. |
| Ravn-Haren G 2018 | AP | Male F344 rats |  | 2.1% or 6.5% | Increase production of short-chain fatty acids and excretion of bile acids to lower cholesterol levels. |
| Zhang XY 2020 | Apple phlorizin | Mice fed with HFD |  | 80 mg/kg | Enhanced GLP-2 secretion and restoration in the gut epithelial barrier. |
| Xiang L 2011 | phloridzin | K6001 yeast strain |  | 3, 10, and 30 µM | SOD and Sir2 have important roles in phloridzin-regulated lifespan extension of yeast, and potentially anti-aging effects for mammalian cells via SIRT1. |
| Wang H 2018 | Apple phlorizin | Male Oregon K wild‐type flies |  | 0.5, 1.0, or 2.0 mg | Treatment with apple phlorizin was found to significantly extend the life span and ameliorate the age-related decline of locomotor function. |
| Wang F 2020 | Apple polyphenols | Chronic ethanol exposure-induced neural injury in rats |  | 140, 280 and 560 mg/kg/d | Apple polyphenols exert a protective effect against chronic ethanol-induced memory impairment by improving the oxidative stress in the hippocampus. |
| Ghumatkar PJ 2015 | Apple phloretin | Scopolamine induced amnesia in mice |  | 2.5, 5, 10mg/kg | Phloretin has nootropic, neuroprotective and neurotrophic activities in SCP-induced memory-impaired mice and hence. |
| Tripathi S 2020 | Apple cider vinegar | Zn with the HFD induced neurotoxicity in mouse brain | STZ-induced cellular toxicity in mouse neuroblastoma cells | Animals: 0.7%; cells:100μM | Apple cider vinegar protects against MTT reduction, tau phosphorylation, amyloid aggregation, alleviation of memory impairment and oxidative stress, and protects against degeneration of cholinergic hippocampal neurons. |
| Tripathi S 2022 | Apple cider vinegar | Swiss albino mice treated with scopolamine | H_2_O_2_ treated Neuro2A cells | Animal:0.7%; Cell:2μM | Increased the SOD and GSH levels, and reduced the MDA level and AchE activity. |
| Tripathi S 2022 | Apple cider vinegar | Zinc-HFD-induced AD mice model |  | 0.7% | Increased the DA, 5-HT, and NA level decreased the MAO-A and MAO-B levels. |
| 4-HNE,4 Hydroxynonenal; 5-HT,5-hydroxytryptamine; 8OHdG,8-hydroxydeoxyguanosine; AchE, acetylcholinesterase ; AE, Apple Extract; ALT, Alanine aminotransferase; AP, Apple Pomace; AS, Atherosclerosis; AST, Aspartate aminotransferase; BAX, Bcl-2 associated X protein; Bcl-, B cell lymphoma 2; BP, Blood Pressure; CAT, Catalase; CGs, Carbonyl groups; Cpt1a, Carnitine Palmitoyltransferase 1A; Cpt1b, Carnitine palmitoyltransferase 1B; CRP, C-reactive protein; CXCL-1, Chemokine ligand 1; Cyto C, Cytochrome c; DNMT3a, DNA methyl transferase 3a; DNMT3b, DNA methyl transferase 3b; ET-1, Endothelin-1; FGF21, Fibroblast Growth Factor 21; FPG, Fasting blood glucose; GLP-2, Glucagon-like peptide 2; GSH-Px, Glutathione peroxidase; GST, Glutathione S-transferase; HDL-C, High-Density Lipoprotein Cholesterol; HFD, High fat diet; IL-1β, Interleukin-1β; IL-6, Interleukin-6; IL-8, Interleukin-8; LBP, LPS-binding protein; LDL-C, Low-Density Lipoprotein Cholesterol; LPO, Lipid peroxidation; LPS, Lipopolysaccharides; MAPK, Mitogen-activated protein kinase; MCP-1, Monocyte chemotactic protein 1; MDA, Malondialdehyde; Mgat-1, Monoacylglycerol O-acyltransferase; MGO, Methylglyoxal; MMP, Matrix metalloproteinase; mTOR, Mammalian target of rapamycin; NF-κB, Nuclear factor-κB; PARP, Poly (ADP-ribose) polymerase; PGC-1α, Peroxisomal proliferator-activated receptor γ coactivator-1α; PGI2, Prostaglandin I2; Pparγ2, Peroxisome proliferator-activated receptor-γ 2; PUFAs, Polyunsaturated fatty acids; RAGE, Receptor For Advanced Glycation End; ROS, Reactive oxygen species; SCFA, Short chain fatty acid; SD, Sprague-Dawley; SOD, Superoxide dismutase; STZ, Streptozotocin; TAM, Amoxifen; TC, Total Cholesterol; TG, Triglyceride; TNF-α, Tumor necrosis factor α; TXA2, Thromboxane A2; UHRF1, Ubiquitinlike with PHD and ring finger domain 1; ULK1, Unc-51-like kinase 1; VCAM-1, Vascular cell adhesion protein 1; VLDL-C, Very Low-Density Lipoprotein Cholesterol; HUVEC, Human umbilical vein endothelial cells. | | | | | |
